# Anticoagulation with osocimab in patients with kidney failure undergoing hemodialysis: a randomized phase 2 trial

**DOI:** 10.1038/s41591-023-02794-7

**Published:** 2024-02-16

**Authors:** Jeffrey I. Weitz, László B. Tankó, Jürgen Floege, Keith A. A. Fox, Deepak L. Bhatt, Ravi Thadhani, James Hung, Ákos F. Pap, Dagmar Kubitza, Wolfgang C. Winkelmayer, CONVERT Investigators

**Affiliations:** 1 Thrombosis and Atherosclerosis Research Institute and McMaster University, Hamilton, Ontario Canada; 2 Clinical Development and Operations, Bayer Consumer Care AG, Basel, Switzerland; 3 Division of Nephrology and Rheumatology, RWTH Aachen University Hospital, Aachen, Germany; 4 Centre for Cardiovascular Sciences, University of Edinburgh, Edinburgh, UK; 5 Mount Sinai Heart, Icahn School of Medicine at Mount Sinai Health System, New York, NY USA; 6 Emory University School of Medicine, Atlanta, GA USA; 7 Clinical Development and Operations, Bayer SA, São Paulo, Brazil; 8 Clinical Data Sciences and Analytics, Bayer AG, Wuppertal, Germany; 9 Clinical Pharmacology, Bayer AG, Wuppertal, Germany; 10 Section of Nephrology, Baylor College of Medicine, Houston, TX USA

**Keywords:** Drug development, End-stage renal disease

## Abstract

Individuals with kidney failure undergoing hemodialysis are at elevated risk for thromboembolic events. Factor (F) XI, which is in the intrinsic pathway of coagulation, is emerging as an attractive target for new anticoagulants that may be safer than existing agents. Osocimab—an inhibitory FXIa antibody—is a potential treatment option for such patients. We conducted a phase 2b, double-blind, placebo-controlled trial, in which 704 participants (448 male, 256 female) with kidney failure undergoing hemodialysis were randomized to receive lower- or higher-dose osocimab or placebo. In total, 686 participants (436 male, 250 female) received treatment for ≤18 months (planned minimal treatment period of 6 months). The co-primary outcomes were clinically relevant bleeding (a composite of major and clinically relevant nonmajor bleeding) and a composite of the incidence of moderate, severe or serious adverse events. Clinically relevant bleeding occurred in 16/232 (6.9%) and 11/224 (4.9%) participants who received lower- and higher-dose osocimab, respectively, and in 18/230 participants (7.8%) who received a placebo. For the composite adverse event endpoint, incidences were 51%, 47% and 43% in the lower-dose osocimab, higher-dose osocimab and placebo groups, respectively. These results suggest that osocimab is associated with a low risk of bleeding and is generally well tolerated in this population; findings that require confirmation in larger trials. ClinicalTrials.gov identifier, NCT04523220.

## Abstract (web summary)

In a phase 2 trial involving patients with kidney failure who were undergoing hemodialysis, treatment with osocimab—an antibody targeting coagulation factor XIa—did not lead to increased rates of clinically relevant bleeding or an increased risk of adverse events as compared to placebo, suggesting the possibility that factor XIa inhibitors may be safer in this patient population than currently available anticoagulants.

## Main

Chronic kidney disease, which affects almost 10% of the global population, is a major cause of morbidity and mortality^1^. Diabetes and hypertension are common causes of kidney failure^2^. Most individuals with kidney failure who are managed with hemodialysis are at risk of major adverse vascular events such as myocardial infarction, stroke and other thromboembolic events^2,3^. Although long-term anticoagulation therapy has the potential to prevent these complications, its use remains understudied because people with kidney failure are also at increased risk of major bleeding^4^. Around one in seven such individuals will experience a major bleed within 3 years of dialysis initiation^5^, and the bleeding risk is further increased, up to tenfold, when people with kidney failure requiring hemodialysis are treated with warfarin^6^. Furthermore, a recent trial comparing warfarin with apixaban for stroke prevention in patients with atrial fibrillation on regular hemodialysis reported comparably high rates of major bleeding (9.7% versus 8.5%)^7^. Therefore, with limited evidence supporting the use of warfarin or direct oral anticoagulants in this patient population, there remains an unmet need for safer anticoagulants for the prevention of thromboembolic events.

Inhibitors of factor XI or its activated form, factor XIa, may be safer than the currently available anticoagulants because factor XI seems to be more important for thrombosis than for hemostasis^8^. Thus, individuals with reduced factor XI levels are at lower risk for thrombosis than those with normal levels^9^, but rarely experience serious bleeding^8^. Conversely, high levels of factor XI increase the risk for thrombosis^8,10,11^. Therefore, factor XI has emerged as a target for potentially safer anticoagulants and may be a particularly attractive target in individuals with kidney failure requiring regular dialysis^3^.

**Fig. 1:**
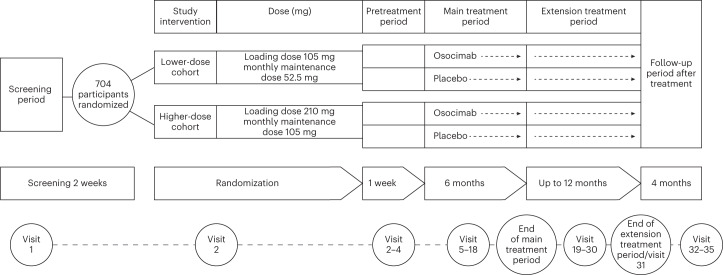
Study design. Overview of the study design. Participants with kidney failure undergoing hemodialysis were randomized to receive lower- or higher-dose osocimab or placebo for ≤18 months. The follow-up period ended 5 months after the last study intervention and 4 months after the end of the main or extension treatment period.

Osocimab is a long-acting, fully human inhibitory antibody directed against factor XIa^12,13^. In a phase 2 dose-finding study in individuals undergoing total knee arthroplasty, a single intravenous dose of osocimab administered before surgery was superior, or after surgery was noninferior, to enoxaparin for prevention of postoperative venous thromboembolism^14^. Furthermore, osocimab was associated with low rates of clinically relevant bleeding, the composite of major and clinically relevant nonmajor bleeding^14^. To address the unmet need for safer anticoagulants for patients with end-stage kidney disease, we conducted the CONVERT trial (ClinicalTrials.gov identifier, NCT04523220) to compare the rates of clinically relevant bleeding with osocimab and placebo in participants with kidney failure who were undergoing hemodialysis (Fig. 1).

## Results

### Patient disposition

**Fig. 2:**
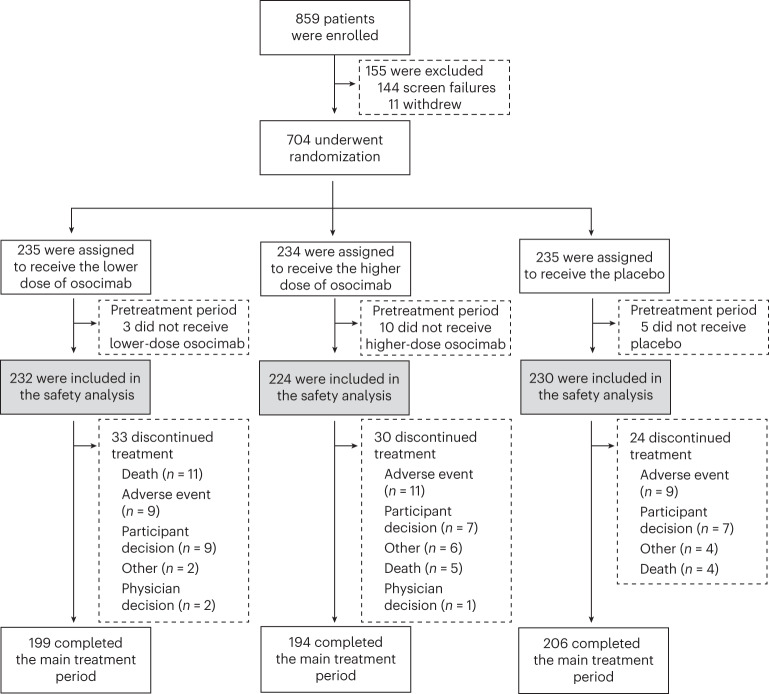
Patient disposition. Summary of patient flow in the phase 2b CONVERT trial.

From August 2020 to April 2021, a total of 704 participants from 147 sites in 19 countries were randomized. After the exclusion of 18 participants who did not receive the study drug, 686 individuals started the main 6-month treatment period and were included in the analyses (Fig. 2). The main treatment period was completed by 199 participants in the lower-dose osocimab group (84.7%), 194 participants in the higher-dose osocimab group (82.9%) and 206 participants in the placebo group (87.7%). The extended treatment period was completed by 178 participants in the lower-dose osocimab group (75.5%), 174 participants in the higher-dose osocimab group (74.4%) and 176 participants in the placebo group (74.9%). The median previous duration on maintenance dialysis was 4.0 years (interquartile range, 2.0–7.2 years). Overall, 249 participants (36.3%) had known atherosclerotic disease and 290 (42.3%) were taking aspirin. The baseline characteristics and median duration of study treatment were similar across groups (Table 1).

**Table 1:** Baseline demographics and clinical characteristics of study participants

Characteristic	Lower-dose osocimab (*n* = 232)	Higher-dose osocimab (*n* = 224)	Placebo (*n* = 230)
Age, median (range) (years)	61 (28–91)	61 (25–90)	60 (24–90)
Age group, *n* (%)			
<60 years	104 (44.8)	103 (46.0)	108 (47.0)
60–75 years	98 (42.2)	84 (37.5)	94 (40.9)
>75 years	30 (12.9)	37 (16.5)	28 (12.2)
Male sex, *n* (%)	143 (61.6)	143 (63.8)	150 (65.2)
Body mass index^a^, *n* (%)			
25–30 kg m^−2^	62 (26.7)	87 (38.8)	93 (40.4)
≥30 kg m^−2^	74 (31.9)	65 (29.0)	62 (27.0)
White, *n* (%)	191 (82.3)	185 (82.6)	185 (80.4)
Geographic region, *n* (%)			
Western Europe	36 (15.5)	37 (16.5)	35 (15.2)
Eastern Europe	133 (57.3)	121 (54.0)	123 (53.4)
Asia Pacific	21 (9.0)	19 (8.4)	19 (8.2)
North America	39 (16.8)	37 (16.5)	37 (16.1)
Australia and Israel	3 (1.2)	10 (4.4)	16 (6.9)
Dialysis duration, median (interquartile range) years	4.05 (2.0–7.2)	4.0 (2.0–7.5)	3.85 (1.8–7.0)
Dialysis access, *n* (%)			
Fistula	191 (82.3)	180 (80.4)	189 (82.2)
Graft	22 (9.5)	20 (8.9)	13 (5.7)
Catheter	19 (8.2)	24 (10.7)	28 (12.2)
Heparin use during dialysis, *n* (%)	221 (95.3)	218 (97.3)	218 (94.8)
Etiology of kidney disease, *n* (%)			
Diabetes	65 (28.0)	53 (23.6)	59 (25.6)
Hypertension	51 (22.0)	67 (30.0)	50 (21.7)
Glomerulonephritis	32 (13.8)	30 (13.4)	33 (14.3)
Pyelonephritis	11 (4.7)	10 (4.5)	10 (4.3)
Polycystic kidney disease	23 (9.9)	23 (10.3)	21 (9.1)
Other	50 (21.5)	41 (18.3)	57 (24.8)
Diabetes, *n* (%)	90 (38.8)	82 (36.6)	87 (37.8)
Hypertension, *n* (%)	213 (91.8)	212 (94.6)	215 (93.5)
Coronary heart disease, *n* (%)	56 (24.1)	61 (27.2)	47 (20.4)
Myocardial infarction, *n* (%)	17 (7.3)	15 (6.7)	11 (4.8)
Peripheral artery disease, *n* (%)	18 (7.8)	23 (10.3)	22 (9.6)
Stroke, *n* (%)	12 (5.1)	18 (7.7)	18 (7.7)
Atrial fibrillation, *n* (%)	15 (6.5)	17 (7.6)	14 (6.1)
Previous cardiovascular event^b^, *n* (%)	37 (15.9)	43 (19.2)	40 (17.4)
Atherosclerosis^c^, *n* (%)	81 (34.9)	86 (38.4)	82 (35.7)
History of venous thromboembolism, *n* (%)	8 (3.4)	10 (4.5)	10 (4.3)
Platelet count, mean (s.d.) × 10^9^ l^−1^	209 (62)	209 (56)	212 (58)
Hemoglobin, median (interquartile range) (mg dl^−1^)	10.9 (10.2–11.8)	11.1 (10.4–11.8)	11.0 (10.4–11.7)
Low-dose aspirin, *n* (%)	97 (41.8)	95 (42.4)	98 (42.6)
Duration of study drug administration, median (interquartile range) (months)	9.1 (7.0–11.7)	9.2 (7.1–11.7)	9.0 (7.1–11.4)
Length of study, *n* (%)			
≤6 months	37 (15.9)	33 (14.7)	26 (11.3)
>6–9 months	76 (32.8)	73 (32.6)	89(38.7)
>9 months	119 (51.3)	118 (52.7)	115 (50.0)

Percentages may not total 100 because of rounding.

^a^Body mass index is the weight in kilograms divided by the square of the height in meters.

^b^Previous cardiovascular events include stroke, transient ischemic attack, myocardial infarction, deep vein thrombosis and pulmonary embolism.

^c^Atherosclerosis was defined as a history of ischemic stroke, transient ischemic attack, unstable angina, myocardial infarction, peripheral artery disease or aortic aneurysm.

### Primary outcomes

Clinically relevant bleeding (the composite of major and clinically relevant nonmajor bleeding) occurred in 16 of 232 participants (6.9%) who received lower-dose osocimab, 11 of 224 (4.9%) who received higher-dose osocimab and 18 of 230 (7.8%) who received placebo (Table 2). Major bleeding occurred in three (1.3%), two (0.9%) and seven (3.0%) participants in the lower- and higher-dose osocimab and placebo groups, respectively. In those over 75 years of age, clinically relevant bleeding per 100 person-years was 20 (90% confidence interval (CI), 7–39) events for lower-dose osocimab, 8 (90% CI, 1–18) events for higher-dose osocimab and 23 (90% CI, 8–44) events for placebo. Corresponding values for individuals on low-dose aspirin were 12 (90% CI, 6–20), 7 (90% CI, 3–13) and 16 (90% CI, 9–25) events per 100 person-years, respectively. Cause-specific hazard ratios and subdistribution hazard ratios for the primary outcomes are provided in Extended Data Tables 1–4.

**Table 2:** Safety and efficacy outcomes

	Lower-dose osocimab (*n* = 232)	Higher-dose osocimab (*n* = 224)	Placebo (*n* = 230)
Clinically relevant bleeding (primary outcome)			
*n* (%)	16 (6.9)	11 (4.9)	18 (7.8)
Events (90% CI) per 100 patient-years	9.7 (6.1–14.1)	6.7 (3.7–10.3)	10.8 (7.0–15.3)
Composite of moderate, severe or serious adverse events (primary outcome)			
*n* (%)	118 (50.9)	106 (47.3)	99 (43.0)
Major bleeding			
*n* (%)	3 (1.3)	2 (0.9)	7 (3.0)
Events (90% CI) per 100 patient-years	1.8 (0.5–3.8)	1.2 (0.2–2.9)	4.2 (2.0–7.1)
Clinically relevant nonmajor bleeding			
*n* (%)	13 (5.6)	9 (4.0)	11 (4.8)
Events (90% CI) per 100 patient-years	7.9 (4.7–11.8)	5.5 (2.9–8.8)	6.6 (3.7–10.2)
Types of major bleeding, *n* (%)			
Gastrointestinal	1 (0.4)	0	1 (0.4)
Urogenital (kidney or bladder)	0	0	2 (0.9)
Intracranial	0	0	1 (0.4)
Eye (intraocular or retinal)	1 (0.4)	0	2 (0.9)
Skin (any vascular access site)	0	2 (0.9)	0
Respiratory (pulmonary)	1 (0.4)	0	0
Procedural	0	0	1 (0.4)
Types of clinically relevant nonmajor bleeding, *n* (%)			
Gastrointestinal	1 (0.4)	1 (0.4)	0
Epistaxis	2 (0.9)	1 (0.4)	2 (0.9)
Urogenital	3 (1.3)	2 (0.9)	1 (0.4)
Skin	4 (1.7)	2 (0.9)	2 (0.9)
Vascular access site	2 (0.9)	4 (1.8)	6 (2.6)
Conjunctival	1 (0.4)	0	0
Major adverse vascular events^a^			
*n* (%)	3 (1.3)	6 (2.7)	7 (3.0)
Events (90% CI) per 100 patient-years	1.7 (0.5–3.7)	3.6 (1.6–6.3)	4.1 (1.9–6.9)
Type of event, *n* (%)			
Myocardial infarction	2 (0.9)	4 (1.8)	4 (1.7)
Ischemic stroke	1 (0.4)	1 (0.4)	2 (0.9)
Major amputation	0	0	1 (0.4)
Systemic embolism	0	1 (0.4)	0
Atherosclerotic subgroup^a^			
*n*/*n* (%)	2/81 (2.5)	2/86 (2.3)	6/82 (7.3)
Events (90% CI) per 100 patient-years	3.7 (0.7–8.7)	3.3 (0.6–7.8)	11.0 (4.8–19.2)
Dialysis circuit clotting, *n* (%)			
Score of 2 or 3	68 (29.3)	61 (27.2)	95 (41.3)
Score of 3	4 (1.7)	4 (1.8)	10 (4.3)
Access thrombosis, *n* (%)	6 (2.6)	8 (3.6)	11 (4.8)
Serious adverse events, *n* (%)			
Serious adverse event	70 (30.2)	64 (28.6)	63 (27.4)
Serious adverse event leading to discontinuation of study drug	8 (3.4)	10 (4.5)	13 (5.7)
All-cause death			
*n* (%)	12 (5.2%)	9 (4.0%)	11 (4.8%)
Events (90% CI) per 100 patient-years	7.0 (4.0–10.6)	5.4 (2.8–8.6)	6.4 (3.6–9.8)
Injection-site reactions, *n* (%)	14 (6.0)	17 (7.6)	1 (0.4)

Incidences are reported by the number of participants having the specific event up to 30 days after the end of study treatment for that individual.

^a^Vascular death (due to myocardial infarction, stroke, pulmonary or systemic embolism), nonfatal myocardial infarction or stroke, major amputation for vascular etiology, acute limb ischemia and symptomatic venous thromboembolism or incidence of thrombosis of arteriovenous fistulas or grafts.

The incidences of moderate, severe or serious adverse events were 51% (*n* = 118), 47% (*n* = 106) and 43% (*n* = 99) in the lower-dose osocimab, higher-dose osocimab and placebo groups, respectively (Table 2).

### Secondary outcomes

**Fig. 3:**
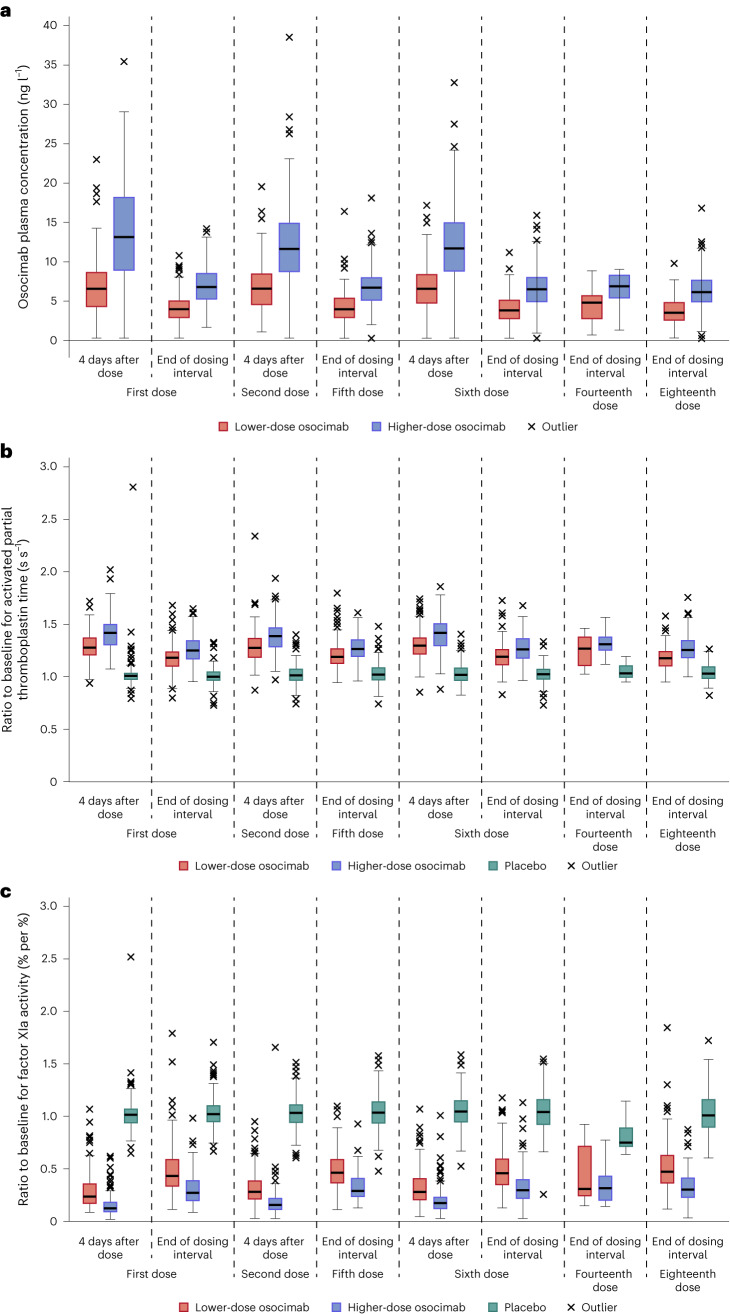
Osocimab plasma concentrations and effects of osocimab treatment on the activated partial thromboplastin time and inhibition of factor XIa activity. **a**, Box plots showing osocimab concentrations. **b**, Activated partial thromboplastin times relative to baseline values. **c**, Inhibition of factor XIa activity relative to baseline values. Medians are indicated by the horizontal lines in the boxes; boxes indicate 25th and 75th percentiles; the vertical lines extend to a maximum distance of 1.5 interquartile ranges; values outside of this range are plotted separately. Analyses are based on the pharmacodynamic analysis set, *n* = 686 (lower-dose osocimab, *n* = 232; higher-dose osocimab, *n* = 224; placebo, *n* = 230).

Prespecified secondary outcomes were osocimab plasma levels, prolongation of the activated partial thromboplastin time and inhibition of factor XIa. There were dose-dependent changes in these variables and their timecourses were comparable from the first to the last measured dose (Fig. 3).

### Safety

Injection-site reactions occurred in 14, 17 and 1 participant in the lower-dose osocimab, higher-dose osocimab and placebo groups, respectively, resulting in study drug discontinuation by two participants from each osocimab group and no participants in the placebo group. Further details regarding the incidence of treatment-related serious adverse events are provided in Extended Data Table 5.

In total, 26 participants received lower- or higher-dose osocimab (*n* = 13 each) within 30 days preceding major surgery or intervention (Extended Data Table 6). The predicted median factor XIa inhibition levels at the time of surgery or intervention were 58% and 69% with the lower and higher osocimab doses, respectively, and no bleeding events were reported during or within 2 weeks of surgery or intervention. Of those randomized to placebo or randomized to osocimab but who did not receive any treatment, 21 participants underwent major surgery or intervention. There was one procedure-related, clinically relevant nonmajor bleed after dialysis catheter removal in the placebo group.

### Exploratory outcomes

For the prespecified exploratory outcomes, major adverse vascular events occurred in 3 of 232 (1.3%) participants who received lower-dose osocimab, 6 of 224 (2.7%) who received higher-dose osocimab and 7 of 230 (3.0%) who received placebo. In participants with known atherosclerotic disease, major adverse vascular events occurred in 4 of 167 (2.4%) individuals who received osocimab and in 6 of 82 (7.3%) who received placebo. No participant experienced symptomatic venous thromboembolism.

Clotting of the dialysis circuit was scored in each participant at every study visit (0, no clot; 1, trace of clot; 2, intermediate between 1 and 3; and 3, fully clotted system necessitating interruption of hemodialysis session). Dialysis circuit clotting scores of 2 or 3 at any visit were reported in 29.3% and 27.2% of participants in the lower- and higher-dose osocimab groups, respectively, and in 41.3% of those in the placebo group. The relative risk of moderate-to-complete clotting at one or more visits was significantly lower in both osocimab groups than in the placebo group (lower dose versus placebo, 0.71 (95% CI, 0.54–0.93; *P* = 0.0085); higher dose versus placebo, 0.66 (95% CI, 0.49–0.87; *P* = 0.0021)).

### Sensitivity analyses

**Extended Data Fig. 1:**
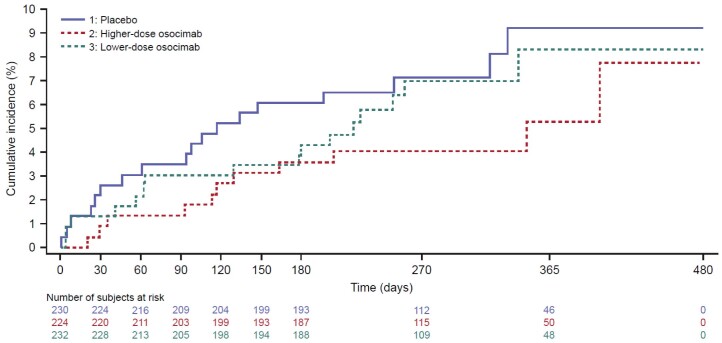
Cumulative incidence risk (Aalen–Johansen) of the time-to-first treatment-emergent composite of major and clinically relevant nonmajor bleeding (primary outcome; safety analysis set). Cumulative incidence (%) = Aalen–Johansen estimates of the cumulative probability for an event, calculated as 100 x (1 minus the Aalen–Johansen estimate of the survival function). Overall study period: Only treatment-emergent events that occurred during the overall study period are considered. Treatment-emergent events are those that occurred after the start of the study treatment and until the last dose of the study treatment plus 30 days.

**Extended Data Fig. 2:**
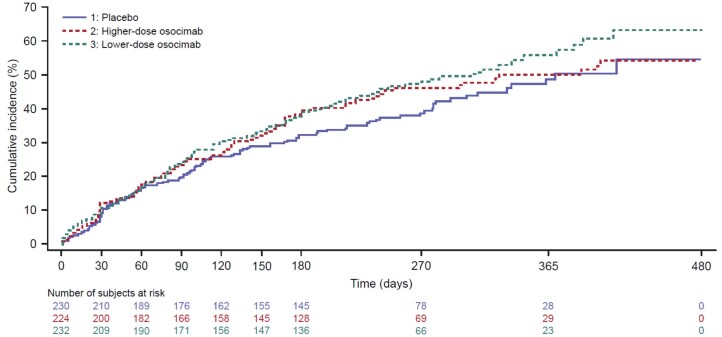
Cumulative incidence risk (Aalen–Johansen) of the time-to-first treatment-emergent moderate, severe, or serious adverse event (primary outcome; safety analysis set). Cumulative incidence (%) = Aalen–Johansen estimates of the cumulative probability for an event, calculated as 100 x (1 minus the Aalen–Johansen estimate of the survival function). Overall study period: Only treatment-emergent events that occurred during the overall study period are considered. Treatment-emergent events are those that occurred after the start of the study treatment and until the last dose of the study treatment plus 30 days.

**Extended Data Fig. 3:**
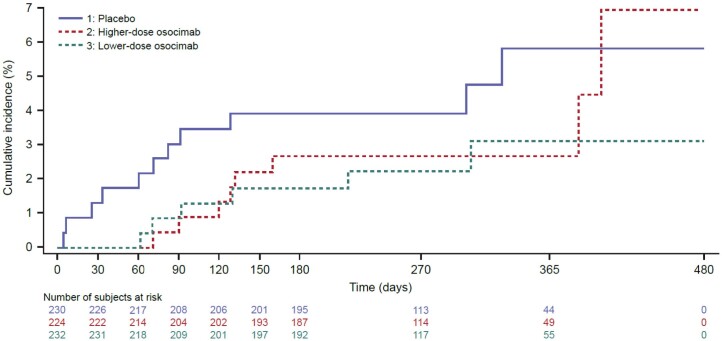
Cumulative incidence risk (Aalen–Johansen) of time-to-first arteriovenous fistula or graft thrombosis (exploratory outcome; safety analysis set). Cumulative incidence (%) = Aalen–Johansen estimates of the cumulative probability for an event, calculated as 100 x (1 minus the Aalen–Johansen estimate of the survival function). Overall study period: Only treatment-emergent events that occurred during the overall study period are considered. Treatment-emergent events are those that occurred after the start of the study treatment and until the last dose of the study treatment plus 30 days.

Aalen–Johansen estimates of the cumulative incidences (that is, the expected proportions of participants with an outcome over time, taking competing risks into account) for the primary outcomes and the exploratory efficacy outcome are provided in Extended Data Figs. 1–3.

For the primary outcome of clinically relevant bleeding, the cumulative incidence risks over the main treatment period were 4.3% (90% CI, 2.48–6.90) for lower-dose osocimab, 3.57% (90% CI, 1.91–6.04) for higher-dose osocimab and 6.09% (90% CI, 3.84–9.04) for placebo. For the second primary outcome of the composite of moderate, severe or serious adverse events over the main treatment period, the cumulative incidence risks were 38.37% (90% CI, 33.10–43.60) for lower-dose osocimab, 38.84% (90% CI, 33.46–44.17) for higher-dose osocimab and 32.17% (90% CI, 27.16–37.28) for placebo.

For the exploratory efficacy outcome of the incidence of arteriovenous fistula or graft thrombosis over the main treatment period, the cumulative incidence risks were 1.7% (90% CI, 0.70–3.61) for lower-dose osocimab, 2.68% (90% CI, 1.29–4.91) for higher-dose osocimab and 3.91% (90% CI, 2.18–6.43) for placebo.

## Discussion

Individuals with kidney failure requiring hemodialysis are at increased risk of bleeding^3,4^. The results of the primary outcomes of this phase 2b trial suggest that, compared with placebo, osocimab does not increase the risk of clinically relevant bleeding or the risk of moderate, severe or serious adverse events. No bleeding events were reported in the small number of osocimab-treated individuals who underwent major surgery or intervention, including the 13 participants who underwent kidney transplantation. Therefore, osocimab seems to be associated with a low rate of clinically relevant bleeding in individuals requiring hemodialysis, which is similar to what was previously reported with osocimab in subjects undergoing elective knee arthroplasty^14^. These findings were observed despite the fact that osocimab prolongs the activated partial thromboplastin time in a dose-dependent manner.

Low event rates precluded exploratory analysis of efficacy regarding the incidence of major adverse vascular events. However, the exploratory analysis of dialysis circuit clotting, which was assessed in all participants, revealed that both doses of osocimab were associated with a significant reduction in the risk of moderate-to-complete dialysis circuit clotting compared with placebo, providing proof of concept that osocimab has antithrombotic effects beyond those of heparin^15^. Extracorporeal circuits activate factor XII and trigger clotting via the intrinsic pathway^16^. By inhibiting factor XIa, which is activated by factor XIIa, osocimab seems to be able to attenuate this process to a greater extent than heparin.

At present, there are no safe and effective anticoagulants for the prevention of thromboembolic events in individuals with kidney failure requiring hemodialysis. Prospective and observational studies have reported little or no benefit and increased bleeding in those receiving oral anticoagulants^17,18^. Two randomized trials comparing vitamin K antagonists with apixaban in individuals with kidney failure undergoing hemodialysis who had atrial fibrillation were able to recruit only 154 and 97 participants, respectively, and were therefore underpowered to yield definitive results^7,19^. The current findings and those of a trial comparing fesomersen—an antisense oligonucleotide that reduces the hepatic synthesis of factor XI—with placebo in individuals with end-stage kidney disease requiring hemodialysis^20^, raise the possibility that factor XI inhibitors may be safer than currently available anticoagulants.

Like the above-mentioned apixaban trials^7,19^, the composite of major and clinically relevant nonmajor bleeding was a primary outcome of the current study. Osocimab is an experimental agent and, as such, adverse events were also included as a primary safety outcome. Stroke and systemic embolism were key secondary outcomes in the apixaban trials because the patients had atrial fibrillation, whereas most of the patients enrolled in the present study did not have atrial fibrillation. Given that the incidences of stroke and systemic embolism are lower in those without atrial fibrillation, a broader range of thromboembolic events was included as exploratory outcomes. Furthermore, because osocimab was compared with a placebo in this trial, the only patients with atrial fibrillation who were eligible for enrollment were those who had been deemed unsuitable for anticoagulation therapy.

Some methodological aspects of our trial require comment. The strengths of the study include the double-blind trial design, the extended duration of treatment and the consistent findings with lower and higher doses of osocimab, which render it unlikely that the reported safety findings reflect a play of chance. Nonetheless, because of the modest sample size, additional studies are needed to assess the safety of osocimab. Finally, although end-stage kidney disease is more prevalent in older than in younger individuals, the prevalence of end-stage kidney disease in those aged 45 to 64 years has increased by 56% over the past 20 years^15^. This may explain why the mean age of participants in the present study was 61 years. Additional studies are needed to assess the safety of osocimab more thoroughly in older subjects.

In summary, compared with placebo, osocimab did not increase the risk of clinically relevant bleeding in individuals with kidney failure requiring hemodialysis. Appropriately powered phase 3 trials are needed to determine whether osocimab reduces the risk of thromboembolic events in this vulnerable and understudied population.

## Methods

Additional information about authors, study sites and investigators, and outcome definitions is provided in the Supplementary Appendix.

### Study design and oversight

This phase 2b, randomized, double-blind, parallel-group trial compared two subcutaneous osocimab dosing regimens with a placebo in individuals with kidney failure requiring hemodialysis (Fig. 1). This multicenter study enrolled participants globally, including in North America, Europe, Asia and Australia.

An academic Steering Committee, in collaboration with the sponsor (Bayer), was responsible for the design and oversight of the study. The sponsor was responsible for data collection, maintenance and analysis. An Institutional Review Board at each participating center approved the protocol and all participants provided informed consent. This study was conducted in accordance with the consensus ethical principles derived from international guidelines including the Declaration of Helsinki and Council for International Organizations of Medical Sciences (CIOMS) International Ethical Guidelines, applicable ICH Good Clinical Practice Guidelines and all applicable laws and regulations.

The Steering Committee was blinded to treatment assignment as were the members of a Central Independent Adjudication Committee, who adjudicated all deaths, suspected bleeds, and cardiovascular or thromboembolic events. An independent Data and Safety Monitoring Committee periodically reviewed trial outcomes and adverse events with the support of an independent Statistical Analysis Center.

The protocol and accompanying documents are available with the full text of this article online.

### Participants

Individuals with kidney failure were eligible for inclusion if they were 18 years of age or older and were undergoing hemodialysis (at least three times per week for a minimum of 9 h per week) and stable for at least 3 months. Patients with atrial fibrillation who were not considered to be candidates for therapeutic anticoagulation by their treating physicians were eligible for inclusion.

Participants were eligible for inclusion if all the following criteria were met:

1. ≥18 years of age
2. End-stage kidney disease undergoing hemodialysis (including hemodiafiltration) for ≥3 months and stable, in the view of the investigator
3. Body weight ≥50 kg
4. Men and women were eligible; contraceptive use should be consistent with local regulations regarding the methods of contraception for those participating in clinical studies
5. Capable of providing signed informed consent

Participants were not eligible for inclusion if any of the following criteria applied:

1. Recent (<6 months before screening) clinically significant bleeding
2. Hemoglobin <9.0 g dl^−1^
3. Platelet count <100 × 10^9^ l^−1^
4. Activated partial thromboplastin time or prothrombin time above the upper limit of normal
5. Hepatic disease associated with alanine aminotransferase over three times the upper limit of normal, or total bilirubin over two times the upper limit of normal with direct bilirubin over 20% of the total
6. Sustained uncontrolled hypertension (diastolic blood pressure ≥100 mmHg and/or systolic blood pressure ≥180 mmHg)
7. Known intracranial neoplasm, arteriovenous malformation or aneurysm
8. Known bleeding disorders
9. Recent (<3 months before screening) thromboembolic event
10. Recent (<3 months before screening) major surgery or scheduled major surgery during study participation
11. Scheduled living donor renal transplant during study participation
12. Persistent heart failure, as classified by the New York Heart Association classification of III or higher
13. Receiving antiplatelet therapy, except acetylsalicylic acid ≤150 mg per day
14. Receiving anticoagulation in therapeutic doses, other than standard anticoagulation during the hemodialysis procedure
15. Life expectancy <6 months
16. Active malignancy requiring treatment during study participation (except nonmelanoma skin cancer or cervical carcinoma in situ)
17. Known hypersensitivity to the investigational drug or to inactive constituents of the study drug
18. Participation in another clinical study with an investigational medicinal product within 30 days or within five half-lives of such, whichever is longer, before randomization and during the study
19. Any other conditions, which, in the opinion of the investigator or sponsor, would render the individual unsuitable for inclusion

### Randomization and study treatment

Participants were randomized between lower-dose osocimab and lower-dose placebo in a ratio of 2:1, and higher-dose osocimab and higher-dose placebo in a ratio of 2:1. Different placebos were needed because a larger volume was administered in the higher-dose group than in the lower-dose group. Participants were centrally assigned using an interactive web-response system and covariate-adaptive randomization. Covariates included geographical region, age, previous major adverse cardiovascular event, dialysis access via catheter, low-dose aspirin use, diabetes and atrial fibrillation. After randomization, there was a 1-week pretreatment period where participants underwent three hemodialysis sessions to establish a baseline for adverse events without treatment. Participants then received osocimab in a lower- or higher-dose regimen or placebo for 6 months in the main treatment period followed by an extension period of up to 12 months, or until the last study participant had completed their 6-month main treatment period. The lower-dose osocimab regimen consisted of a 105 mg loading dose followed by a monthly maintenance dose of 52.5 mg, and the higher-dose regimen consisted of a 210 mg loading dose followed by a monthly maintenance dose of 105 mg. A matching placebo was provided in identical-appearing vials. Treatments were administered subcutaneously in the abdomen no more than 1 h before dialysis. Heparin was administered as per usual during the dialysis sessions. The maximum duration of treatment was 18 months, and the trial was stopped when the last randomized participant completed 6 months of treatment.

### Study outcomes

#### Primary outcomes

The primary outcomes were (1) clinically relevant bleeding, namely the composite of major and clinically relevant nonmajor bleeding, and (2) the composite of moderate or severe adverse events and serious adverse events. Bleeding was classified as major if it was overt and associated with a decrease in hemoglobin of 2 g dl^−1^ or more; necessitated transfusion of two or more units of blood; occurred in a critical area or organ; or contributed to death. Overt bleeding not meeting these criteria, but that necessitated medical examination or intervention, or had clinical consequences, was classified as clinically relevant nonmajor bleeding. If neither set of criteria was met, bleeding was classified as minor.

Prespecified secondary outcomes were assessments of the change from baseline in osocimab concentration and key pharmacodynamic parameters, namely activated partial thromboplastin time and inhibition of factor XIa. Osocimab concentrations were measured by immunoassay, activated partial thromboplastin times were measured using C.K. Prest—a kaolin activator (Diagnostica Stago)—and factor XIa inhibition was quantified using a proprietary fluorogenic assay. Assays were conducted after in vitro neutralization of heparin to eliminate the potential effects of heparin in these assays.

#### Exploratory outcomes

Prespecified exploratory outcomes included the incidence of major adverse vascular events, the composite of vascular death due to myocardial infarction, stroke or pulmonary or systemic embolism; nonfatal myocardial infarction or stroke; major amputation of vascular etiology; acute limb ischemia and symptomatic venous thromboembolism.

Additionally, the incidence of arteriovenous fistula or graft thrombosis, and clotting of the dialysis circuit was assessed at every study visit as a prespecified exploratory outcome. Dialysis circuit clotting in the filter and air trap was assessed at the end of the hemodialysis procedure by study personnel blinded to treatment allocation. Clotting scores were assigned using a visual scoring system (0, no clot; 1, trace of clot; 2, intermediate between 1 and 3; and 3, fully clotted system necessitating interruption of hemodialysis session).

### Statistical analysis

The major objectives of this study were to document the incidences of major and clinically relevant nonmajor bleeding for assessment of safety and arterial and venous thrombotic events for exploration of efficacy. There were no formal hypotheses. All analyses were descriptive and there was no formal a priori sample size calculation. Based on historic operational experience, of approximately 600 participants assigned randomly to study intervention, 555 participants would be expected to complete the main treatment period (assuming a true incidence rate of 15 participants lost to follow up per 100 participant years across all groups). Data were analyzed using SAS base v.9.4 (SAS/STAT v.14.3).

The efficacy and safety analyses were performed in the safety analysis set, defined as all randomized participants who received at least one dose of study medication. Safety and efficacy outcomes included all events that occurred during study treatment and up to 30 days after the last study drug administration. Outcomes were described by incidence proportions and cause-specific incidence rates per 100 person-years and their 90% CIs. Cause-specific hazard ratios were estimated with Cox proportional hazards model, subdistribution hazard ratios were estimated by Fine–Gray subdistribution hazards model. Because of the exploratory nature of these analyses, no multiplicity adjustment was performed. The number of participants who had a clotting score of 2 or 3 (moderate-to-complete dialysis circuit clotting) at one or more visits during the overall treatment period was compared between treatment groups by calculating relative risks and 95% CIs, where CIs were determined with the SAS procedure PROC FREQ by inverting two separate one-sided exact tests that were based on the score statistic (post hoc analysis; Farrington–Manning score statistic)^21,22^.

### Sensitivity analyses

The cumulative incidence functions for the event-of-interest as well as the associated competing events together with the corresponding CIs were estimated for each treatment arm using Aalen–Johansen estimators. The competing events for the primary endpoints were death and premature discontinuation of exposure to assigned treatment. The cumulative incidences were estimated for time-to-event endpoints by Aalen–Johansen estimators with the competing event. The differences of the Aalen–Johansen estimators between high-dose osocimab and placebo and low-dose osocimab and placebo are presented with 90% CIs.

### Reporting summary

Further information on research design is available in the Nature Portfolio Reporting Summary linked to this article.

## Online content

Any methods, additional references, Nature Portfolio reporting summaries, source data, extended data, supplementary information, acknowledgements, peer review information; details of author contributions and competing interests; and statements of data and code availability are available at 10.1038/s41591-023-02794-7.

### Supplementary information

Supplementary Information Supplementary appendix including author affiliations, Steering Committee, Data Monitoring Committee, IECs and IRBs, Clinical Events Adjudication Committee, study sites and investigators, and outcome definitions.

Reporting Summary

## Figures and Tables

**Extended Data Table 1:**
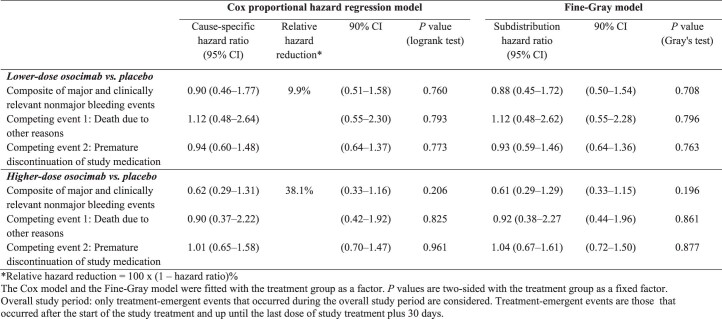
Cause-specific hazard ratios and subdistribution hazard ratios for treatment-emergent primary outcome (composite of major and clinically relevant nonmajor bleeding) and competing events (safety analysis set)

**Extended Data Table 2:**
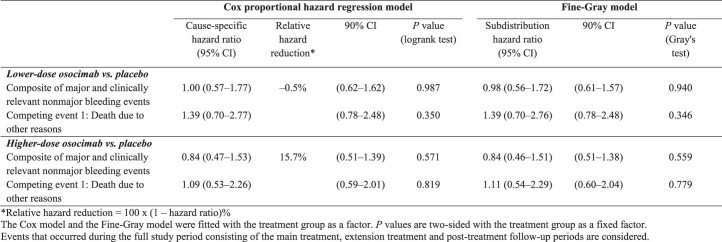
Cause-specific hazard ratios and subdistribution hazard ratios for primary outcome (composite of major bleeding and clinically relevant nonmajor bleeding) and competing events (safety analysis set)

**Extended Data Table 3:**
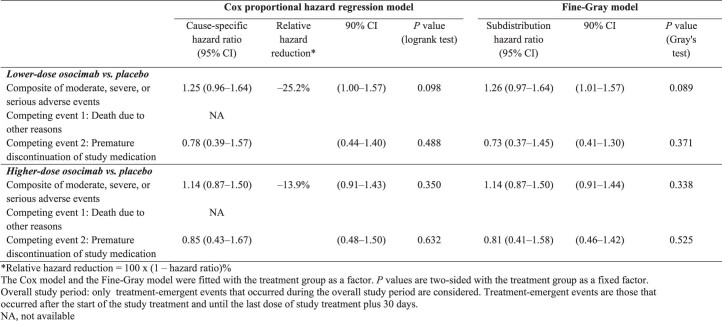
Cause-specific hazard ratios and subdistribution hazard ratios for the treatment-emergent composite of moderate, severe, or serious adverse events (primary outcome) and competing events (safety analysis set)

**Extended Data Table 4:**
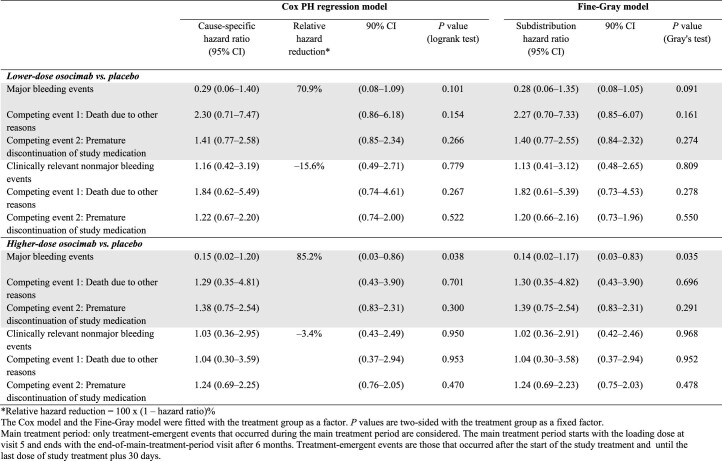
Cause-specific hazard ratios and subdistribution hazard ratios for treatment-emergent major bleeding events, clinically relevant nonmajor bleeding events, and competing events (safety analysis set)

**Extended Data Table 5:**
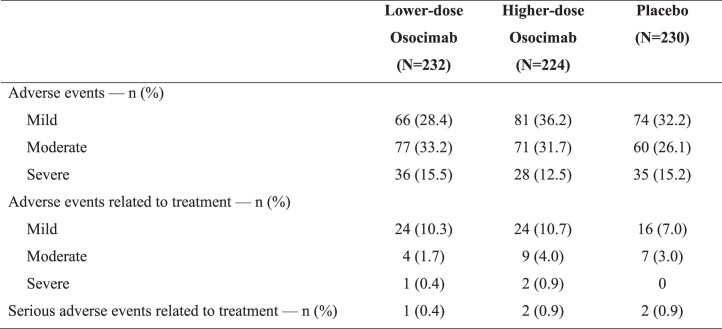
Summary of Adverse Events

**Extended Data Table 6:**
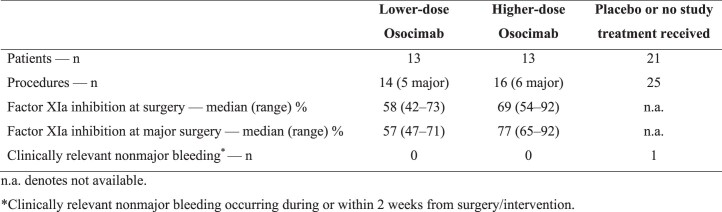
Surgical Interventions Within One Month After Administration of Study Drug, Extent of Factor XI Inhibiton, and Associated Bleeding Outcomes*

## Data Availability

Availability of the data underlying this publication will be determined according to Bayer’s commitment to the EFPIA/PhRMA ‘Principles for responsible clinical trial data sharing.’ This pertains to the scope, timepoint and process of data access. As such, Bayer commits to sharing clinical trial data at the patient and study level upon request from qualified research personnel, as well as protocols from clinical trials for medicines and indications approved in the United States and European Union (necessary for conducting legitimate research). This applies to data on new medicines and indications that have been approved by the European Union and United States regulatory agencies on or after 1 January 2014. Interested researchers can use www.vivli.org to request access to anonymized patient-level data and supporting documents from clinical studies to conduct further research that can help advance medical science or improve patient care. Information about the Bayer criteria for listing studies and other relevant information is provided in the member section of the portal. Data access to anonymized patient-level data, protocols and clinical study reports will be granted after approval by an independent scientific review panel. Data will be made available within 6 months after signing the Data Use Agreement to researchers who provide a methodologically sound proposal. Bayer is not involved in the decisions made by the independent review panel. Bayer will take all necessary measures to ensure that patient privacy is safeguarded.
