# Supplementary material for: Anticoagulation with osocimab in patients with kidney failure undergoing hemodialysis: a randomized phase 2 trial
Source: Nat Med. 2024 Feb 16;30(2):435–42. doi: 10.1038/s41591-023-02794-7 (PMC10878964; doi:10.1038/s41591-023-02794-7)
Supplement: Supplementary file 1 — Supplementary appendix including author affiliations, Steering Committee, Data Monitoring Committee, IECs and IRBs, Clinical Events Adjudication Committee, study sites and investigators, and outcome definitions. [file 41591_2023_2794_MOESM1_ESM.pdf]

nature medicine

Article

https://doi.org/10.1038/s41591-023-02794-7

Anticoagulation with osocimab in patients with kidney failure undergoing hemodialysis: a randomized phase 2 trial

In the format provided by the authors and unedited

Supplementary Appendix

This appendix has been provided by the authors to give readers additional information about their work.

Supplement to: A Phase II Randomized Trial of Osocimab in Patients with Kidney Failure Undergoing Hemodialysis

Table of contents

Author Affiliations 2
Steering Committee 2
Data Monitoring Committee 3
Clinical Events Adjudication Committee 4
Study Sites and Investigators 5
IEC and IRB list 12
Outcome Definitions 33
Safety outcomes 33
Clinically relevant bleeding 33
Assessment of Arteriovenous Access Bleeding 34
Adverse events 35
Efficacy Outcomes 35
Major adverse vascular events: 35
Arteriovenous fistula or graft thrombosis: 36
Dialysis circuit clotting 36
Known atherosclerosis 36

Author Affiliations

Thrombosis and Atherosclerosis Research Institute and McMaster University (J.I.W.) – both in Hamilton, Canada; Clinical Development and Operations, Bayer Consumer Care AG, Basel, Switzerland (L.B.T.); Division of Nephrology and Rheumatology, RWTH Aachen University Hospital, Aachen, Germany (J.F.); Centre for Cardiovascular Sciences, University of Edinburgh, Edinburgh, UK (K.F.); Mount Sinai Heart, Icahn School of Medicine at Mount Sinai Health System, New York, New York (D.L.B.); Emory University School of Medicine, Atlanta, Georgia (R.T.); Section of Nephrology, Baylor College of Medicine, Houston, Texas (W.C.W.) – all in the USA; Clinical Development and Operations, Bayer SA, Sao Paulo, Brazil (J.H.); Clinical Data Sciences and Analytics (A.F.P.) and Clinical Pharmacology (D.K.), Bayer AG- both in Wuppertal, Germany.

Steering Committee

Steering Committee members

Name	Function in Steering Committee	Specialty	Voting Status
Ravi Thadhani	Chair	Nephrologist	Voting member
Wolfgang C. Winkelmayer	Member	Nephrologist	Voting member
Jürgen Floege	Member	Nephrologist	Voting member
Jeffrey I. Weitz	Member	Hematologist	Voting member
Keith Fox	Member	Cardiologist	Voting member
Deepak L. Bhatt	Member	Cardiologist	Voting member

Permanent Sponsor Members

Name	Function in Steering Committee	Voting Status
László B. Tankó	Global Clinical Leader	Voting member
Claudia Rahner	Study Statistician	Nonvoting member
Kate Benson	Clinical Project Manager	Nonvoting member
Ariane Dihlmann	Global Safety Leader	Nonvoting member
Dilsad Durmus	Study Manager	Nonvoting member
James Hung	Study Medical Expert	Nonvoting member

Beside the permanent members, ad hoc guests could be invited.

Data Monitoring Committee

Name	Expertise	Affiliation
Sam Schulmann (Chair)	Hematologist	McMaster University<br>237 Barton Street East<br>Hamilton, ON L8L 2X2<br>Canada
Peter Verhamme	Vascular Medicine,<br>Thrombosis<br>and Haemostasis	University Hospitals Leuven<br>Herestraat 49<br>3000 Leuven<br>Belgium
Tara I-Hsin Chang	Nephrologist	Stanford University<br>Department: Medicine -<br>Med/Nephrology<br>MC 5851<br>777 Welch Road Suite DE, Room D100<br>Standford, California 94305-5851<br>USA
Ashkan Shoamanesh	Neurologist	Population Health Research Institute<br>(PHRI)<br>237 Barton Street East<br>Hamilton, ON L8L 2X2<br>Canada
Johannes Mann	Nephrologist	KfH Kuratorium für Dialyse und<br>Nierentransplantation e.V.<br>KfH-Nierenzentrum<br>Isoldenstraße 15<br>80804 München - Schwabing
Christopher Bull Granger	Cardiologist	Duke University Scholl of Medicine<br>200 Morris Street<br>Durham, NC 27701<br>USA
Robin Roberts	Biostatistician	Professor Emeritus,<br>Department of Health Research<br>Methods, Evidence, and Impact<br>McMaster University<br>McMaster Health Sciences Centre<br>1200 Main St W<br>Hamilton, Ontario, Canada<br>L8N 3Z5

Clinical Events Adjudication Committee

Name*	Role
**Principal investigator**	
Renato Lopes, M.D.	CEC PI: phase 1–2 reviewer
**Faculty reviewers**	
Brad Kolls, M.D.	Neuro: phase 1–2 reviewer
David Kong, M.D.	Phase 1–2 reviewer
Dedrick Jordan, M.D.	Neuro: phase 1–2 reviewer
Rob Harrison, M.D.	Phase 1–2 reviewer
Robert McGarrah, M.D.	Phase 1–2 reviewer
Shreyansh Shah, M.D.	Phase 1–2 reviewer
Thomas Povsic, M.D.	Phase 1–2 reviewer
Patrick Pun, M.D.	Phase 1–2 reviewer

*Adjudicators are faculty members of the Duke Clinical Research Institute.

Study Sites and Investigators

Country	Site	Principal Investigator	Participants enrolled (no.)
Australia	John Hunter Hospital	Dr Alastair Gillies	2
	Eastern Health Integrated Renal Service	Dr Matthew Roberts	1
	Sydney Adventist Hospital	Dr Muh Geot Wong	2
	Westmead Hospital	Dr Vincent Lee	1
Austria	Klinik Landstraße - Krankenhaus Rudolfstiftung	Prof Bernhard Ludvik	2
	Klinikum Klagenfurt am Wörthersee	Prof Dr Markus Peck-Radosavljevic	1
	Universitätsklinikum AKH Wien	Prof Rainer Oberbauer	10
	Privatklinik Wehrle-Diakonissen	Prof Dr Raimund Weitgasser	1
Belgium	UZ Leuven Gasthuisberg	Prof Bjorn Meijers	7
	Epicura	Dr Frédéric Debelle	3
	Imeldaziekenhuis - St-Elisabethkliniek	Dr Wim Lemahieu	5
	AZ Nikolaas Campus Sint-Niklaas	Dr Johan De Meester	7
	CHU de Liege | CHR Citadel - Department of Nephrology	Dr Luc Radermacher	1
	AZ St-Lucas Campus St-Lucas	Dr Céline Seghers	2
Bulgaria	UMHAT Kanev AD	Dr Radka Karagyozova	12
	MHAT Sveti Ivan Rilski 2003	Prof Pencho Simeonov	7
	MHAT Haskovo	Dr Vladimir Popiliev	5
	Multiprofile Hospital for Active Treatment Hristo Botev AD	Prof Valeri Simeonov	7
	MHAT Shumen AD	Dr Nedyu Nedev	12
Czechia	Krajska Nemocnice Liberec a.s.	Dr Miroslav Ryba	7
	Privamed s.r.o.	Dr Jan Wirth	2
	Fresenius Nephro Care s.r.o. - Sokolov	Dr Jiri Vlasak	5
	Fakultni nemocnice Ostrava	Dr Ivo Valkovsky	2
Greece	General Hospital of Athens LAIKO	Prof Ioannis Boletis	1

	General Hospital of Thessaloniki Papageorgiou	Dr Dorothea Papadopoulou	4
	Hippokration General Hospital of Thessaloniki	Dr Aikaterini Papagianni	3
	University General Hospital of Larissa	Prof Ioannis Stefanidis	4
	HIPPOKRATION General Hospital of Athens	Dr Dimitrios Petras	5
	Ioannina University General Hospital	Prof Evangelia Ntounousi	2
	General Hospital of Arta	Dr Chariklia Gouva	11
Hungary	Keszthelyi Korhaz	Dr Laszlo Kovats	3
	Bajai Szent Rokus Korhaz	Dr Katalin Magyar	9
	Csongrad Megyei Egeszsegugyi Ellato Kozpont,Hodmezovasa rhely	Dr Ildiko Csaszar	14
	Fresenius Medical Care Egeszsegugyi Kft.	Dr Botond Csiky	11
	Fresenius Medical Care Egeszsegugyi Kft.	Dr Erzsebet Ladanyi	4
	Fresenius Medical Care Egeszsegugyi Kft.	Dr Peter Kevei	8
	Magyar Honvedseg Egeszsegugyi Kozpont	Dr Karoly Schneider	7
Israel	Health Corporation of Galilee Medical Center	Dr Etty (Esther) Kruzel-Davila<br>Dr Khaled Khazim	2
	Poriya Medical Center | Nephrology and Hypertension Dept.	Dr Evgeny Farber	8
	Barzilai Medical Center | Nephrology & Hypertension Dept.	Prof Yoram Yagil	9
	Clalit Health Services Rabin Medical Center- Beilinson Campus	Prof Benaya Rozen-Zvi	4
	Shamir Medical Center (Assaf Harofeh)	Prof Ilia Beberashvili	8
	Lady Davis Carmel Medical Center	Dr Victor Frajewicki	4
	Rabin Medical Center, HaSharon (Golda) Campus	Prof Benaya Rozen-Zvi	5

Italy	Fondazione Policlinico Universitario Agostino Gemelli IRCCS	Prof Giuseppe Grandaliano	4
	A.O.U. Careggi	Dr Calogero Lino Cirami	3
	IRCCS Istituti Clinici Scientifici Maugeri SpA SB	Prof Ciro Esposito	3
	A.O.U. di Parma	Prof Enrico Fiaccadori	2
	A.O.U. di Bologna Policlinico S.Orsola Malpighi	Prof Gaetano La Manna	4
	IRCCS Ospedale Policlinico San Martino	Dr Maura Ravera	3
	AUSL-IRCCS di Reggio Emilia	Dr Maria Cristina Gregorini	1
Japan	Saiyu Clinic	Dr Noriyuki Kato	3
	Kikuchi Medical Clinic	Dr Hiroshi Kikuchi	6
	Akagaki Clinic	Dr Yoji Akagaki	6
	Saint Hill Hospital	Dr Zenzo Fujii	11
	Katta General Hospital	Dr Sadayoshi Ito	8
		Dr Yorihiro Akamatsu	
	Yabuki Hospital	Dr Ikuto Masakane	6
	Ohishi Naika Clinic	Dr Akira Ohishi	4
	Kyoto Station Takeda Dialysis Clinic	Dr Tetsuro Yoshioka	5
	Nagaoka Red Cross Hospital	Dr Hajime Yamazaki	2
	Ibaraki Seinan Medical Center Hospital	Dr Tadashi Iitsuka	2
	Takemura Medical Nephro Clinic	Dr Katsumi Takemura	2
	Akebono Clinic	Dr Motoko Tanaka	2
	Futakotamagawaekima e clinic	Dr Toshikazu Imai	2
	Tsuchiura Beryl Clinic	Dr Masaru Mori	6
Lithuania	LUHS Kauno Hospital (Josvainiu str.)	Dr Gintautas Gumbrevicius	6
Netherlands	Academisch Medisch Centrum (AMC)	Dr Liffert Vogt	3
	Catharina Ziekenhuis	Dr Stijn Konings	2
	Deventer Ziekenhuis	Dr Cornelius Doorenbos	1
		Dr Daan de Boer	
	Maasstad Ziekenhuis	Dr Rene van den Dorpel	3

	Albert Schweitzer Ziekenhuis, Locatie Dordwijk	Dr Jeroen van der Net	3
	Gelre Ziekenhuizen	Dr Jos Barendregt	3
Poland	Stacja Dializ W-wa Mangalia	Dr Marta Serwanska-Swietek	6
	Wojewodzki Szpital Zespolony	Prof Andrzej Jaroszynski	4
	NZOZ Nowy Szpital w Swieciu Sp. z o.o.	Dr Andrzej Brymora	8
	Stacja Dializ Olkusz	Dr Pawel Kozminski	7
	Stacja Dializ Zyrardow	Dr Krzysztof Wroblewski	5
Portugal	Eurodial Obidos (DaVita)	Prof Andre Weigert	1
	CHLO - Hospital Santa Cruz	Dr Rita Birne	4
	NephroCare APDP	Dr Patricia Branco	6
	Pluribus Dialise - Cascais (DaVita)	Dr Jorge Dickson	4
	CHMT - Hospital Rainha Santa Isabel - Servico de Nefrologia	Dr Hernani Goncalves	2
	Caledial - Centro de Hemodialise de Gaia	Prof Serafim Guimaraes	3
	Diaverum Aveiro	Dr Lidia Santos	2
	Eurodial Leiria (DaVita)	Dr Joana Silva Costa	4
	Pluribus Dialise - Sacavem (DaVita)	Dr Artur Mendes	4
	SPD - Amadora (Diaverum Amadora)	Dr Hugo Neves Silva	4
Russian Federation	Sverdlovsk Regional Clinical Hospital #1	Dr Elena Boretskaya	4
	City Hospital #31	Dr Galina Timokhovskaya	19
	LLC Fresenius medical care Kuban	Dr Lubov Eremeeva	5
	LLC B. Brown Avitum Russland Clinics	Dr Anastasiya Sabodash	27
	LLC Dialysis center	Dr Natalia Novoseltseva	5
	LLC Dialysis center	Dr Irina Selezneva	4
	LLC Yaroslavl dialysis center	Dr Tatyana Abissova	19
	Republican Hospital n.a. V.A. Baranov	Dr Aleksandr Zuev	5
	Kupchinsky center for outpatient dialysis	Dr Alexey Tutin	7

	Chelyabinsk Regional Clinical Hospital	Dr Lyudmila Zhuravleva	8
	City Clinical Hospital #1 Orenburg	Dr Alexandr Selyutin	20
Spain	Corporació Sanitària Parc Taulí	Dr José Antonio Ibeas López	3
	Hospital Clínico Universitario de Valencia	Dr Jose Gorriz Teruel	1
	Hospital Clínico Universitario de Santiago de Compostela	Dr Candido Diaz Rodriguez<br>Dra Dolores Güimil Carbajal	1
	Hospital Universitario Dr Peset	Dr Pablo Molina Vila	5
	Hospital Althaia, Xarxa Assistencial de Manresa	Dr Josep Maria Galcerán Gui	5
	Hospital Clinico Universitario San Carlos | Nefrologia	Dr José Antonio Herrero Calvo	4
	Hospital Universitari Germans Trias i Pujol	Dr Fredzzia Graterol Torres	3
Turkey	Kocaeli Universitesi Tip Fakultesi	Dr Necmi Eren	8
	Hacettepe Universitesi Tip Fakultesi	Prof Mustafa Arici	3
	Turk Bobrek Vakfi Memorial Hizmet Hastanesi	Dr Funda Yalcin	3
	Erciyes Universitesi Tip Fakultesi	Dr Ismail Kocyigit	4
	Inonu Universitesi Tip Fakultesi	Mr Bayram Berktas<br>Prof Idris Sahin	4
	Adnan Menderes Universitesi Tip fakultesi	Prof Yavuz Yenicerioglu	2
	Sisli Hamidiye Etfal Egitim ve Arastirma Hastanesi	Prof Abdulkadir Unsal	1
Ukraine	Brovarskaya multidisciplinary clinical hospital	Dr Andrii Shen	7
	Kyiv City Center of Nephrology and Dialysis	Dr Iryna Dudar	12
	Medical center LLC (Fresenius medical care Ukraine)	Dr Volodymyr Novakivskyy	13

	Regional Clinical Hospital - Ivano- Frankivsk	Dr Oleg Legun	9
	Kharkiv regional Clinical Centre of urology and nephrology	Dr Sergiy Kolupayev	7
	State Institution “Institute of Nephrology” NAMS of Ukraine	Dr Mykola Kolesnyk	10
	Lutsk city clinical hospital	Dr Tetyana Trigub	3
	Mykolaiv Regional Clinical Hospital	Dr Tetyana Kostynenko	6
	Vinnitsa Regional Clinical Hospital im. N.I. Pirogov	Dr Nataliia Pyvovarova	7
	Zaporizhzhia Regional Clinical Hospital	Dr Svitlana Korneyeva	13
	Transcarpatian Regional Clinical Hospital	Dr Vasyl Stryzhak	16
	Dnepropetrovsk regional hospital n.a. I. I. Mechnikov	Dr Olga Galushchak	12
	Poltava Regional Clinical Hospital n.a. M.V. Skliphosovskyi	Dr Galyna Osinnia	5
USA	Davita Hospital Hill Dialysis	Dr Ahmed Awad	21
	Fresenius Kidney Care - Brawley	Dr Ramin Berenji	6
	Fresenius Kidney Care Evergreen Park	Dr Paul Crawford	22
	DaVita Butler Farm Dialysis	Dr Olayiwola Ayodeji	4
	ARA Holyoke Dialysis Center	Dr Michael Germain	2
	Fresenius Kidney Care South Dialysis	Dr Marwan Omar Kaskas	8
	Waterloo Dialysis	Dr Judith Betts	2
	ARA Augusta	Dr Devesh Patel	2
	Fresenius Kidney Care - La Mesa	Dr George Fadda	12
	DaVita South Valley Dialysis	Dr Kenneth Kleinman	3
	Renal Medicine Associates	Dr Jayant Kumar	1
	Liberty Dialysis- Caldwell	Dr Arnold Silva	3
	ARA Plantation Dialysis, LLC	Dr Hakan Toka	3

	DaVita Greater Waterbury Dialysis	Dr Sina Raissi	4
	North America Research Institute - Azusa	Dr Aamir Jamal	5
	Van Buren Dialysis Center	Dr Mohammed Khan	14
	East L.A. Dialysis Center	Dr Carlos Meza	9
	FMC San Ysidro	Dr Jill Meyer	2
	Fresenius Kidney Care Kearny Mesa	Dr Dylan Steer	10
	Durham Nephrology Associates, Pa	Dr Amarnath Kathresal	4
	Fresenius Kidney Care Baton Rouge Mancuso Lane	Dr Michael Roppolo	3
	Fresenius Kidney Care Sugar Land	Dr Charles Henry	1
	Liberty Dialysis St. George	Dr Andrew Patchett	2
	Fresenius North Austin Dialysis Center	Dr Eileen Cook	3
	Fresenius Kidney Care – Mission Bend (FMCNA #3971)	Dr Marializa Bernardo	8
	Queen’s Dialysis Unit	Dr Nitin Bhasin	15

IEC and IRB list

T Number	TC Country / Region	TC Centre Name	TC Centre Location Address	TC Centre Location-Province	TU Centre Name	TU Centre Location Address	TU Centre Location-Province	TU Number	TU Reference
20115	Australia	Gold Coast Hospital	Gold Coast Hospital Gold Coast Hospital and Health Service HREC Office for Research Governance and Development Level 2 Block E – Pathology and Education 1 Hospital Boulevard 4215 Southport	Queensland (AU-QLD)					
					Eastern Health Integrated Renal Service	Eastern Health Integrated Renal Service, Office of Research and Ethics, Eastern Health, Level 4, 5 Arnold St, 3128, Box Hill, AU-VIC	Victoria (AU-VIC)	40006	Not Available
					John Hunter Hospital	John Hunter Hospital, Hunter New England Human Research Ethics Committee, Lookout Road, 2305, New Lambton,		40003	Not Available
					Sydney Adventist Hospital	Sydney Adventist Hospital, Research and Governance Office, Adventist HealthCare Limited, 185 Fox Valley Road, 2076, Wahroonga, AU-NSW	New South Wales (AU-NSW)	40007	Not Available
					Westmead Hospital	Westmead Hospital, Sydney West Area Health Service Human Research Ethics Committee, Nepean Campus, Court Building, Ground Floor, PO Box 63, 2751, Penrith, AU-NSW	New South Wales (AU-NSW)	40009	Not Available
		Westmead Hospital	Westmead Hospital HREC Clinical Sciences Hawkesbury Rd. 2145 Westmead	New South Wales (AU-NSW)					
					Eastern Health Integrated Renal Service	Eastern Health Integrated Renal Service, Office of Research and Ethics, Eastern Health, Level 4, 5 Arnold St, 3128, Box Hill, AU-VIC	Victoria (AU-VIC)	40006	Not Available

T Number	TC Country / Region	TC Centre Name	TC Centre Location Address	TC Centre Location-Province	TU Centre Name	TU Centre Location Address	TU Centre Location-Province	TU Number	TU Reference
					John Hunter Hospital	John Hunter Hospital, Hunter New England Human Research Ethics Committee, Lookout Road, 2305, New Lambton,		40003	Not Available
					Sydney Adventist Hospital	Sydney Adventist Hospital, Research and Governance Office, Adventist HealthCare Limited, 185 Fox Valley Road, 2076, Wahroonga, AU-NSW	New South Wales (AU-NSW)	40007	Not Available
					Westmead Hospital	Westmead Hospital, Sydney West Area Health Service Human Research Ethics Committee, Nepean Campus, Court Building, Ground Floor, PO Box 63, 2751, Penrith, AU-NSW	New South Wales (AU-NSW)	40009	Not Available
	Austria	Medizinische Universität Graz	Medizinische Universität Graz Ethikkommission der Medizinischen Universität Graz Auenbruggerplatz 2 8036 Graz	Steiermark (AT-STM)					
	Belgium	UZ Antwerpen	UZ Antwerpen Ethisch Comité Drie Eikenstraat 655 2650 EDEGEM						
					AZ Nikolaas Campus Sint-Niklaas	AZ Nikolaas Campus Sint-Niklaas, Ingang Moerlandstraat, Moerlandstraat 1, 9100, SINT-NIKLAAS,		28005	Not Available
					AZ St-Lucas Campus St-Lucas	AZ St-Lucas Campus St-Lucas, Ethisch Comité, Groenebriel 1, 9000, GENT,		28007	Not Available

T Number	TC Country / Region	TC Centre Name	TC Centre Location Address	TC Centre Location-Province	TU Centre Name	TU Centre Location Address	TU Centre Location-Province	TU Number	TU Reference
					CHR de la Citadelle	CHR de la Citadelle, CHR de la Citadelle, Comité Ethique, Boulevard du 12e de Ligne 1, 4000, LIEGE,		28006	Not Available
					Epicura	Epicura, Epicura, Site de Baudour, Comité Ethique, Rue Louis Caty 136, 7331, BAUDOUR,		28002	Not Available
					Imeldaziekenhuis - St-Elisabethkliniek	Imeldaziekenhuis - St-Elisabethkliniek, Ethisch Comité, Imeldalaan 9 , 2820, BONHEIDEN,		28003	Not Available
					UZ Leuven Gasthuisberg	UZ Leuven Gasthuisberg, Ethische Commissie Onderzoek UZ/KU Leuven, Herestraat 49, 3000, LEUVEN,		28001	28001
	Bulgaria	Ethical Committee for Multicenter Trials	Ethical Committee for Multicenter Trials Damyan Gruev Str. 8 1303 Sofia						
	Czechia	Eticka Komise	Eticka Komise Fakultni nemocnice Brno Jihavska 20 62500 Brno						
					EK Fakultni nemocnice Ostrava	EK Fakultni nemocnice Ostrava, ETICKA KOMISE Fakultni nemocnice Ostrava, 17 Listopadu 1790, 708 52, Ostrava,		38008	Not Available

T Number	TC Country / Region	TC Centre Name	TC Centre Location Address	TC Centre Location-Province	TU Centre Name	TU Centre Location Address	TU Centre Location-Province	TU Number	TU Reference
					EK Fresenius Medical Care – DS, s.r.o	EK Fresenius Medical Care – DS, s.r.o, ETICKA KOMISE Fresenius Medical Care – DS, s.r.o, Evropska 423/178, 160 00, Praha 6,		38005	Not Available
					Eticka Komise	Eticka Komise, Fakultni nemocnice Brno, Jihavska 20, 62500, Brno,		38004	Not Available
					Eticka komise pri Krajske nemocnicni Liberec a.s.	Eticka komise pri Krajske nemocnicni Liberec a.s., Husova 10, Krajska nemocnice Liberec a.s., 460 63, Liberec,		38002	Not Available
	Greece	National Ethics Committee	National Ethics Committee 284 Mesogeion Avenue 15562 Cholargos/Athens						
	Hungary	Medical Research Council EC for Clinical Pharmacology	Medical Research Council EC for Clinical Pharmacology Alkotmany utca 25. 1054 Budapest						
					B-A-Z MK es EOK IKEB	B-A-Z MK es EOK IKEB, Szentpeteri kapu 72-76., 3526, Miskolc,		46006	Not Available
					Bajai Szent Rokus Korhaz IKEB	Bajai Szent Rokus Korhaz IKEB, Rokus u. 10., 6500, Baja,		46003	Not Available

T Number	TC Country / Region	TC Centre Name	TC Centre Location Address	TC Centre Location-Province	TU Centre Name	TU Centre Location Address	TU Centre Location-Province	TU Number	TU Reference
					Csongrad Megyei Eu Ellato Kozpont, IKEB	Csongrad Megyei Eu Ellato Kozpont, IKEB, Dr. Imre J. u. 2., 6800, Hodmezovasarhely,		46004	Not Available
					Keszthelyi Korhaz IKEB	Keszthelyi Korhaz IKEB, Ady Endre u. 2., 8360, Keszthely,		46002	Not Available
					MH Eu Kozpont Intezmenyi es Region. Kutatasetikai Bizottsag	MH Eu Kozpont Intezmenyi es Region. Kutatasetikai Bizottsag, Robert Karoly korut 44., 1134, Budapest,		46009	Not Available
					PTE-OEC IKEB	PTE-OEC IKEB, Pecsi Tudomanyegyetem Klinikai Kozpont, Regionalis Kutatasetikai Bizottsaga, Rakoczi u. 2., 7624, Pecs,		46005	Not Available
					Regionalis, Intezmenyi Tudomanyos es Kutatasetikai Bizottsag	Regionalis, Intezmenyi Tudomanyos es Kutatasetikai Bizottsag, Ulloi ut 93. fsz. 2., 1091, Budapest,		46007	Not Available
	Israel				Barzilai Medical Center	Barzilai Medical Center, Ethics Committee, Hahistadrout St 2, 7830604, Ashkelon,		39003	Not Available
					Clalit Health Services Rabin Medical Center-Beilinson Campus	Clalit Health Services Rabin Medical Center-Beilinson Campus, Ethical Committee, 39 Jabotinski Street, 4941492, Petah Tikva,		39005	39005*

T Number	TC Country / Region	TC Centre Name	TC Centre Location Address	TC Centre Location-Province	TU Centre Name	TU Centre Location Address	TU Centre Location-Province	TU Number	TU Reference
								39008	39005-1
					Health Corporation of Galilee Medical Center	Health Corporation of Galilee Medical Center, Ethical Committee, Ben Zvi Avenue, Road 89, P.O.B 21, 2210001, Nahariya,		39001	Not Available
					Lady Davis Carmel Medical Center	Lady Davis Carmel Medical Center, Ethics Committee, 7, Michal Street, 3436212, Haifa,		39007	Not Available
					Shamir Medical Center (Assaf Harofeh)	Shamir Medical Center (Assaf Harofeh), Ethical Committee, Pavilion 206, 7030000, Zrifin,		39006	Not Available
					The Baruch Padeh Medical Center, Poriya	The Baruch Padeh Medical Center, Poriya, Ethics Committee, M.P. Lower Galilee, 1528001, Tiberias,		39002	Not Available
	Italy				A.O.U. Careggi	A.O.U. Careggi, CEAVCE: Comitato Etico di Area Vasta Centro Regione Toscana, Presidente: Dr. Marco Matucci Cerinic, A.O.U. Careggi - Pad. 3 Didattica - Piano 2 St.219, Largo Brambilla, 3, 50134, Firenze, IT_TO	Toscana (IT_TO)	22002	Not Available
					A.O.U. di Bologna Policlinico S.Orsola Malpighi	A.O.U. di Bologna Policlinico S.Orsola Malpighi, CE-AVEC: Comitato Etico Area Vasta Emilia Centro, Presidente: Dr. Primiano Iannone, Policlinico S.Orsola-Malpighi, Via Albertoni, 15, 40138, Bologna, IT-RE	Emilia-Romagna (IT-RE)	22006	Not Available

T Number	TC Country / Region	TC Centre Name	TC Centre Location Address	TC Centre Location-Province	TU Centre Name	TU Centre Location Address	TU Centre Location-Province	TU Number	TU Reference
					A.O.U. di Parma	A.O.U. di Parma, CE-AVEN: Comitato Etico Area Vasta Emilia Nord, Presidente: Prof. Roberto Giuseppe Grilli, Policlinico di Modena, Direzione Assistenza Farmaceutica, Ingresso n.3, Piano Terra, Via del Pozzo, 71, 41124, Modena, IT-RE	Emilia-Romagna (IT-RE)	22004	Not Available
					ASST Lodi	ASST Lodi, COMITATO ETICO MILANO AREA 1, Presidente: Prof. Anna Maria Di Giulio, Ospedale Luigi Sacco, Via G.B. Grassi, 74, 20157, Milano, IT_LO	Lombardia (IT_LO)	22011	Not Available
					AUSL-IRCCS di Reggio Emilia	AUSL-IRCCS di Reggio Emilia, CE-AVEN: Comitato Etico Area Vasta Emilia Nord, Presidente: Prof. Roberto Giuseppe Grilli, AUSL–IRCCS di Reggio Emilia, Via Vertoiba, 10a, 42124, Reggio Emilia, IT-RE	Emilia-Romagna (IT-RE)	22010	Not Available
					Fondazione Policlinico Universitario Agostino Gemelli IRCCS	Fondazione Policlinico Universitario Agostino Gemelli IRCCS, COMITATO ETICO FONDAZIONE POLICLINICO UNIVERSITARIO AGOSTINO GEMELLI IRCCS UNIV.CATTOLICA, Presidente: Prof. Andrea Bacigalupo, Policlinico Universitario A. Gemelli - Ex Collegio Ioanneum - 1° piano stanza 221, Largo A. Gemelli, 8, 00168, Roma, IT_LA	Lazio (IT_LA)	22001	Not Available
					IRCCS Istituti Clinici Scientifici Maugeri SpA SB	IRCCS Istituti Clinici Scientifici Maugeri SpA SB, COMITATO ETICO ISTITUTI CLINICI SCIENTIFICI MAUGERI SPA - IRCCS, Presidente: Dr. Marcello Imbriani, ICS Maugeri Spa-SB, Via S. Maugeri, 4, 27100, Pavia, IT_LO	Lombardia (IT_LO)	22003	Not Available
					IRCCS Ospedale Policlinico San Martino	IRCCS Ospedale Policlinico San Martino, COMITATO ETICO REGIONALE DELLA LIGURIA, Presidente: Avv. Paolo Gianatti, Ospedale Policlinico San Martino, Largo R. Benzi, 10, 16132, Genova, IT_LI	Liguria (IT_LI)	22009	Not Available
		Fondazione Policlinico Universitario Agostino Gemelli IRCCS	Fondazione Policlinico Universitario Agostino Gemelli IRCCS COMITATO ETICO FONDAZIONE POLICLINICO UNIVERSITARIO AGOSTINO GEMELLI IRCCS UNIV.CATTOLICA Presidente: Prof. Andrea Bacigalupo Policlinico Universitario A. Gemelli - Ex Collegio Ioanneum - 1° piano stanza 221 Largo A. Gemelli, 8 00168 Roma	Lazio (IT_LA)					

T Number	TC Country / Region	TC Centre Name	TC Centre Location Address	TC Centre Location-Province	TU Centre Name	TU Centre Location Address	TU Centre Location-Province	TU Number	TU Reference
					A.O.U. Careggi	A.O.U. Careggi, CEAVCE: Comitato Etico di Area Vasta Centro Regione Toscana, Presidente: Dr. Marco Matucci Cerinic, A.O.U. Careggi - Pad. 3 Didattica - Piano 2 St.219, Largo Brambilla, 3, 50134, Firenze, IT_TO	Toscana (IT_TO)	22002	Not Available
					A.O.U. di Bologna Policlinico S.Orsola Malpighi	A.O.U. di Bologna Policlinico S.Orsola Malpighi, CE-AVEC: Comitato Etico Area Vasta Emilia Centro, Presidente: Dr. Primiano Iannone, Policlinico S.Orsola-Malpighi, Via Albertoni, 15, 40138, Bologna, IT-RE	Emilia-Romagna (IT-RE)	22006	Not Available
					A.O.U. di Parma	A.O.U. di Parma, CE-AVEN: Comitato Etico Area Vasta Emilia Nord, Presidente: Prof. Roberto Giuseppe Grilli, Policlinico di Modena, Direzione Assistenza Farmaceutica, Ingresso n.3, Piano Terra, Via del Pozzo, 71, 41124, Modena, IT-RE	Emilia-Romagna (IT-RE)	22004	Not Available
					ASST Lodi	ASST Lodi, COMITATO ETICO MILANO AREA 1, Presidente: Prof. Anna Maria Di Giulio, Ospedale Luigi Sacco, Via G.B. Grassi, 74, 20157, Milano, IT_LO	Lombardia (IT_LO)	22011	Not Available
					AUSL-IRCCS di Reggio Emilia	AUSL-IRCCS di Reggio Emilia, CE-AVEN: Comitato Etico Area Vasta Emilia Nord, Presidente: Prof. Roberto Giuseppe Grilli, AUSL–IRCCS di Reggio Emilia, Via Vertoiba, 10a, 42124, Reggio Emilia, IT-RE	Emilia-Romagna (IT-RE)	22010	Not Available
					Fondazione Policlinico Universitario Agostino Gemelli IRCCS	Fondazione Policlinico Universitario Agostino Gemelli IRCCS, COMITATO ETICO FONDAZIONE POLICLINICO UNIVERSITARIO AGOSTINO GEMELLI IRCCS UNIV.CATTOLICA, Presidente: Prof. Andrea Bacigalupo, Policlinico Universitario A. Gemelli - Ex Collegio Ioanneum - 1° piano stanza 221, Largo A. Gemelli, 8, 00168, Roma, IT_LA	Lazio (IT_LA)	22001	Not Available
					IRCCS Istituti Clinici Scientifici Maugeri SpA SB	IRCCS Istituti Clinici Scientifici Maugeri SpA SB, COMITATO ETICO ISTITUTI CLINICI SCIENTIFICI MAUGERI SPA - IRCCS, Presidente: Dr. Marcello Imbriani, ICS Maugeri Spa-SB, Via S. Maugeri, 4, 27100, Pavia, IT_LO	Lombardia (IT_LO)	22003	Not Available

T Number	TC Country / Region	TC Centre Name	TC Centre Location Address	TC Centre Location-Province	TU Centre Name	TU Centre Location Address	TU Centre Location-Province	TU Number	TU Reference
					IRCCS Ospedale Policlinico San Martino	IRCCS Ospedale Policlinico San Martino, COMITATO ETICO REGIONALE DELLA LIGURIA, Presidente: Avv. Paolo Gianatti, Ospedale Policlinico San Martino, Largo R. Benzi, 10, 16132, Genova, IT_LI	Liguria (IT_LI)	22009	Not Available
	Japan				Adachi Kyosai Hospital	Adachi Kyosai Hospital, Institutional Review Board, 1- 36-8 Yanagihara, 120-0022, Adachi-ku, JP-13	Tokyo (JP-13)	20004	Not Available
								20008	Not Available
					Nagaoka Red Cross Hospital	Nagaoka Red Cross Hospital, Institutional Review Board, 2-297-1 Chiaki, 940-2085, Nagaoka, JP-15	Niigata (JP-15)	20014	Not Available
					Review Board of Human Rights and Ethics for Clinical Studies	Review Board of Human Rights and Ethics for Clinical Studies, Institutional Review Board, 13-2 Ichiban-cho, 102-0082, Chiyoda-ku, JP-13	Tokyo (JP-13)	20001	Not Available
								20015	Not Available
					Sone Clinic	Sone Clinic, Institutional Review Board, 3-32-8 Shinjuku, 160-0022, Shinjuku-ku, JP-13	Tokyo (JP-13)	20019	Not Available

T Number	TC Country / Region	TC Centre Name	TC Centre Location Address	TC Centre Location-Province	TU Centre Name	TU Centre Location Address	TU Centre Location-Province	TU Number	TU Reference
								20020	Not Available
					Sugiura Clinic	Sugiura Clinic, Institutional Review Board, 4-4-16-30… Honcho, 332-0012, Kawaguchi, JP-11	Saitama (JP-11)	20002	Not Available
								20005	Not Available
								20006	Not Available
								20007	Not Available
								20018	Not Available
								20021	Not Available

T Number	TC Country / Region	TC Centre Name	TC Centre Location Address	TC Centre Location-Province	TU Centre Name	TU Centre Location Address	TU Centre Location-Province	TU Number	TU Reference
					Takeda Hospital Group Institutional Review Board	Takeda Hospital Group Institutional Review Board, 28- 1, Ishidamoriminamicho, Fushimi-ku, 601-1495, Kyoto, JP-26	(JP-26)	20009	Not Available
	Lithuania	Lithuanian Bioethics Committee	Lithuanian Bioethics Committee Algirdo str. 31 LT-03219 Vilnius						
					Lithuanian Bioethics Committee	Lithuanian Bioethics Committee, Algirdo str. 31, LT-03219, Vilnius,		57002	57002
	Netherlands	St. Antonius Ziekenhuis	St. Antonius Ziekenhuis MEC-U Koekoekslaan 1, 3435 CM NIEUWEGEIN						
	Poland	Komisja Bioetyczna przy OIL	Komisja Bioetyczna przy OIL ul. Powstancow Warszawy 11 Bydgoszcz						
	Portugal	CEIC	CEIC Parque da Saude de Lisboa Av. do Brasil, no. 53-Pav. 17-A 1749-004 Lisboa	Lisboa (PT- 11)					
					CEIC	CEIC, Parque da Saude de Lisboa, Av. do Brasil, no. 53-Pav. 17-A, 1749-004, Lisboa, PT-11	Lisboa (PT- 11)	42001	42001

T Number	TC Country / Region	TC Centre Name	TC Centre Location Address	TC Centre Location-Province	TU Centre Name	TU Centre Location Address	TU Centre Location-Province	TU Number	TU Reference
								42002	42002
								42003	42003
								42004	42004
								42005	42005
								42006	42006
								42007	42007
								42008	42008

T Number	TC Country / Region	TC Centre Name	TC Centre Location Address	TC Centre Location-Province	TU Centre Name	TU Centre Location Address	TU Centre Location-Province	TU Number	TU Reference
								42009	42009
								42011	42011
	Russian Federation				ArteMed Assistans	ArteMed Assistans, 55, litera A, Pionerskaya st., office 1n/1, 197110, St. Petersburg,		51015	Not Available
					Chelyabinsk Regional Clinical Hospital	Chelyabinsk Regional Clinical Hospital, 70, Vorovskogo st., 454076, Chelyabinsk,		51016	Not Available
					City Clinical Hospital #1 Orenburg	City Clinical Hospital #1 Orenburg, 23, Gagarina prospekt, 460040, Orenburg,		51017	Not Available
					City Hospital #31	City Hospital #31, Local Ethical Committee, 3, Dinamo prospekt, 197110, St. Petersburg,		51004	Not Available
					LLC Dialysis center	LLC Dialysis center, Ethical Committee, 35, Valovaya street, 115054, Moscow,		51008	Not Available

T Number	TC Country / Region	TC Centre Name	TC Centre Location Address	TC Centre Location-Province	TU Centre Name	TU Centre Location Address	TU Centre Location-Province	TU Number	TU Reference
								51009	Not Available
					LLC Fresenius medical care Kuban	LLC Fresenius medical care Kuban, Local Ethical Committee, 22, Cherkasskaya str., 350029, Krasnodar,		51006	Not Available
					LLC Yaroslavl dialysis center	LLC Yaroslavl dialysis center, Local Ethical Committee, 7, Yakovlevskaya str., 150062, Yaroslavl,		51010	Not Available
					Republican Hospital n.a. V.A. Baranov	Republican Hospital n.a. V.A. Baranov, 3, Pirogova st., 185019, Petrozavodsk,		51014	Not Available
					St. Petersburg SBHI "Municipal Mariinskaya	St. Petersburg SBHI "Municipal Mariinskaya, 56, Liteiny prospect, 191014, St. Petersburg,		51007	Not Available
					Sverdlovsk Regional Clinical Hospital #1	Sverdlovsk Regional Clinical Hospital #1, 185, Volgogradskaya st., 620102, Yekaterinburg,		51002	Not Available
	Spain	Hospital Clínico Universitario de Valencia	Hospital Clínico Universitario de Valencia Secretaría Comite Etico de Investigacion Clínica Fundacion Hospital Clínico Universitario de Valencia Avda. Blasco Ibañez, 17 46010 Valencia	Valencia (ES- V)					

T Number	TC Country / Region	TC Centre Name	TC Centre Location Address	TC Centre Location-Province	TU Centre Name	TU Centre Location Address	TU Centre Location-Province	TU Number	TU Reference
	Turkey	Kocaeli Universitesi Etik Kurulu	Kocaeli Universitesi Etik Kurulu Kocaeli Universitesi Umuttepe yerleskesi Universite etik kurulu 41380 Izmit/Kocaeli						
	Ukraine				Brovarskaya multidisciplinary clinical hospital	Brovarskaya multidisciplinary clinical hospital, Ethical Committee, 14, Shevchenko st, 07400, Brovary,		53001	Not Available
					Dnepropetrovsk regional hospital n.a. I. I. Mechnikov	Dnepropetrovsk regional hospital n.a. I. I. Mechnikov, 14 Sobornaya str.,, 49005, Dnipro,		53016	Not Available
					Kharkiv regional Clinical Centre of urology and nephrology	Kharkiv regional Clinical Centre of urology and nephrology, Ethical Committee, 195 Moskovsky av., 61037, Kharkiv,		53006	Not Available
					Kyiv City Center of Nephrology and Dialysis	Kyiv City Center of Nephrology and Dialysis, Ethical Committee, 26, Str. Petra Zaporozhtsia, 02660, Kyiv,		53002	Not Available
					Lutsk city clinical hospital	Lutsk city clinical hospital, Ethical Committee, 13 Vidrodzhenia av., 43024, Lutsk,		53009	Not Available
					Medical center LLC " Fresenius medical care Ukraine»	Medical center LLC " Fresenius medical care Ukraine», Ethics Committee of Medical center LLC Fresenius medical care Ukraine, 32 Dakhnovskaya street / 2 Meditsinsky lane, 18009, Cherkasy,		53003	Not Available

T Number	TC Country / Region	TC Centre Name	TC Centre Location Address	TC Centre Location-Province	TU Centre Name	TU Centre Location Address	TU Centre Location-Province	TU Number	TU Reference
					Mykolaiv Regional Clinical Hospital	Mykolaiv Regional Clinical Hospital, Ethical Committee, 1 Kyivska str., 54058, Mykolaiv,		53011	Not Available
					Poltava Regional Clinical Hospital n.a. M.V. Skliphosovskyi	Poltava Regional Clinical Hospital n.a. M.V. Skliphosovskyi, Ethical Committee, Shevchenko str 23, 36011, Poltava,		53018	Not Available
					Regional Clinical Hospital - Ivano-Frankivsk	Regional Clinical Hospital - Ivano-Frankivsk, Ethics Committee of Regional clinical hospital, Fedkovycha str 91, 76000, Ivano-Frankivsk,		53005	Not Available
					State Institution “Institute of Nephrology” NAMS of Ukraine	State Institution “Institute of Nephrology” NAMS of Ukraine, Kyiv City Nephrology Center, 26 Petra Zaporozhtsya str, 02225, Kyiv,		53008	Not Available
					Transcarpatian Regional Clinical Hospital	Transcarpatian Regional Clinical Hospital, Ethical Committee, 22 Kapushanska str., 88018, Uzhgorod,		53015	Not Available
					Vinnitsa Regional Clinical Hospital im. N.I. Pirogov	Vinnitsa Regional Clinical Hospital im. N.I. Pirogov, Ethical Committee, 46 Pirogova Street, 21018, Vinnytsia,		53013	Not Available
					Zaporizhzhia Regional Clinical Hospital	Zaporizhzhia Regional Clinical Hospital, Local Ethical Committee, 10 Orirhivske shosse, 69600, Zaporizhzhya,		53014	Not Available

T Number	TC Country / Region	TC Centre Name	TC Centre Location Address	TC Centre Location-Province	TU Centre Name	TU Centre Location Address	TU Centre Location-Province	TU Number	TU Reference
	United States of America	Advarra IRB	Advarra IRB 6100 Merriweather Drive Suite 600 21044 Columbia	Maryland (US-MD)					
					Advarra IRB	Advarra IRB, 6100 Merriweather Drive, Suite 600, 21044, Columbia, US-MD	Maryland (US-MD)	14001	Not Available
								14002	Not Available
								14003	Not Available
								14004	Not Available
								14005	Not Available
								14006	Not Available

T Number	TC Country / Region	TC Centre Name	TC Centre Location Address	TC Centre Location-Province	TU Centre Name	TU Centre Location Address	TU Centre Location-Province	TU Number	TU Reference
								14007	Not Available
								14008	Not Available
								14009	Not Available
								14010	Not Available
								14011	Not Available
								14013	Not Available
								14014	Not Available

T Number	TC Country / Region	TC Centre Name	TC Centre Location Address	TC Centre Location-Province	TU Centre Name	TU Centre Location Address	TU Centre Location-Province	TU Number	TU Reference
								14015	Not Available
								14016	Not Available
								14017	Not Available
								14018	Not Available
								14019	Not Available
								14020	Not Available
								14022	Not Available

T Number	TC Country / Region	TC Centre Name	TC Centre Location Address	TC Centre Location-Province	TU Centre Name	TU Centre Location Address	TU Centre Location-Province	TU Number	TU Reference
								14023	Not Available
								14024	Not Available
								14025	Not Available
								14027	Not Available
								14030	Not Available
								14031	Not Available
								14032	Not Available

T Number	TC Country / Region	TC Centre Name	TC Centre Location Address	TC Centre Location-Province	TU Centre Name	TU Centre Location Address	TU Centre Location-Province	TU Number	TU Reference
								14033	Not Available
								14038	Not Available

Outcome Definitions

Safety outcomes

Clinically relevant bleeding

Randomized participants were evaluated for the occurrence of bleeding events after signing informed consent until the end of the post-treatment follow-up. All bleeding events (other than the normal bleeding from arteriovenous graft or fistula post-dialysis) were reported as adverse events and submitted to the Central Independent Adjudication Committee.

Clinically relevant bleeding, the composite of major and clinically relevant nonmajor bleeding, was the primary safety outcome. The Central Independent Adjudication Committee classified bleeding events in alignment with the International Society on Thrombosis and Haemostasis definitions.[1]

Major bleeding was defined as symptomatic bleeding with one of the following:

- bleeding that contributed to death
- symptomatic bleeding in a critical area or organ, such as intracranial, intraspinal, intraocular, retroperitoneal, intraarticular or pericardial, or intramuscular with compartment syndrome
- bleeding causing a fall in hemoglobin level of 2 g/dL (1.24 mmol/L) or more, or leading to transfusion of two or more units of whole blood or packed red blood cells.

Clinically relevant nonmajor bleeding events were classified as any sign or symptom of hemorrhage (e.g., more bleeding than would be expected for a clinical circumstance, including bleeding found by imaging alone) that did not fit the criteria for the International

Society on Thrombosis and Haemostasis definition of major bleeding, but met at least one of the following criteria:

- requiring medical intervention by a healthcare professional
- led to hospitalization or increased level of care
- prompting a face-to-face evaluation (i.e., not a telephone or electronic communication).

Bleeding after dialysis from arteriovenous graft or fistulas was expected and was only reported as an adverse event and submitted to the Central Independent Adjudication Committee if there was a change in the pattern, duration, or intensity of bleeding, or if exceptional measures were taken for hemostasis beyond normal, relative to post-dialysis bleeding that was observed in that participant over (approximately) the prior 4 weeks.

Assessment of Arteriovenous Access Bleeding

The severity of access site bleeding was assessed approximately 15 minutes after removal of the dialysis needle(s) from the arteriovenous access according to the following categories:

- “0” – no access bleeding
- “1” – slow oozing
- “2” – overt bleeding.

If the arteriovenous access exhibited overt bleeding 15 minutes after removal of the dialysis needle(s) (score of 2), the investigator could consider a reduction in the dose of heparin (i.e., unfractionated or low-molecular-weight heparin) for the subsequent dialysis sessions during the treatment and follow-up periods.

If a dialysis catheter was used, the extent of arteriovenous access bleeding was not determined.

Recommendations for bleeding and surgery management can be found in Section 8.2.2.2 of the clinical study protocol.

Adverse events

Adverse events were reported directly by the participant or by the participant's caregiver or legally authorized representative from the time of providing informed consent to the last follow-up visit. All serious adverse events had to be reported within 24 hours.

Using the case report form, investigators or qualified designees were responsible for recording and documenting reported events that met the adverse event or serious adverse event definitions, including details regarding the intensity of the event and its relationship to the study treatment. Furthermore, the investigator or designee followed up on any adverse events that were serious, considered related to the study treatment or procedures, or that led to treatment or study discontinuation. The responsibility to actively monitor adverse events and serious adverse events did not extend beyond the end of the study. However, if an investigator learned of a serious adverse event (including death) that occurred after the end of the study and considers it to be reasonably related to study intervention or participation, the investigator had to notify the sponsor.

Efficacy Outcomes

Major adverse vascular events:

Major adverse vascular events included the following, the definitions for which are detailed in Chapter 5 of the Clinical Events Committee Adjudication Manual:

- death due to myocardial infarction, ischemic stroke, pulmonary embolism, noncentral nervous system systemic embolism, and undetermined – presumed cardiovascular death
- nonfatal stroke (ischemic stroke or undetermined type)
- nonfatal myocardial infarction
- major amputation of vascular etiology
- acute limb ischemia
- symptomatic venous thromboembolism
- systemic embolism.

Arteriovenous fistula or graft thrombosis:

Access site thrombosis was defined as the absence of a bruit or thrill and/or the inability to initiate dialysis successfully via the fistula or graft.

Dialysis circuit clotting

Clotting in the dialysis circuit was assessed using a semiquantitative scale. After every dialysis session, the dialysis nurse (or another qualified individual) assigned clotting scores as follows:

- "0" – clean filter and no visible clots in the drip chamber/air trap
- "1" – traces of coagulation in the filter and/or in the drip chamber/air trap
- "2" – intermediate state between "1" and "3"
- "3" – fully clotted extracorporeal system resulting in an interruption of the hemodialysis session.

Adequate anticoagulation was defined as a semiquantitative clotting scale score of 1 or less.

Known atherosclerosis

Known atherosclerosis was defined as the composite of the following criteria:

(1) any surrogate of coronary artery disease (history of myocardial infarction, angina pectoris, coronary artery bypass graft surgery, percutaneous coronary intervention, coronary artery stent insertion, aortic aneurysm, coronary artery disease)

or

(2) any surrogate of cerebrovascular disease (history of ischemic stroke, transient ischemic attack, or carotid endarterectomy)

or

(3) any surrogate of peripheral artery disease (history of peripheral artery disease, amputation, peripheral artery bypass, or vascular stent insertion)

REFERENCE

1. Kaatz S, Ahmad D, Spyropoulos AC, Schulman S, Subcommittee on Control of Anticoagulation. Definition of clinically relevant non-major bleeding in studies of anticoagulants in atrial fibrillation and venous thromboembolic disease in non-surgical patients: communication from the SSC of the ISTH. J Thromb Haemost. 2015;13(11):2119-26
