# Supplementary material for: Anticoagulation with osocimab in patients with kidney failure undergoing hemodialysis: a randomized phase 2 trial
Source: Nat Med. 2024 Feb 16;30(2):435–42. doi: 10.1038/s41591-023-02794-7 (PMC10878964; doi:10.1038/s41591-023-02794-7)
Supplement: Supplementary file 2 — Reporting Summary [file 41591_2023_2794_MOESM2_ESM.pdf]

nature portfolio

Corresponding author(s): Jeffrey Weitz MD
Last updated by author(s): 21 Dec 2023

Reporting Summary

Nature Portfolio wishes to improve the reproducibility of the work that we publish. This form provides structure for consistency and transparency in reporting. For further information on Nature Portfolio policies, see our Editorial Policies and the Editorial Policy Checklist.

Please do not complete any field with "not applicable" or n/a. Refer to the help text for what text to use if an item is not relevant to your study.
For final submission: please carefully check your responses for accuracy; you will not be able to make changes later.

Statistics

For all statistical analyses, confirm that the following items are present in the figure legend, table legend, main text, or Methods section.

n/a	Confirmed	
☐	☑	The exact sample size (n) for each experimental group/condition, given as a discrete number and unit of measurement
☐	☑	A statement on whether measurements were taken from distinct samples or whether the same sample was measured repeatedly
☐	☑	The statistical test(s) used AND whether they are one- or two-sided<br>*Only common tests should be described solely by name; describe more complex techniques in the Methods section.*
☐	☑	A description of all covariates tested
☑	☐	A description of any assumptions or corrections, such as tests of normality and adjustment for multiple comparisons
☐	☑	A full description of the statistical parameters including central tendency (e.g. means) or other basic estimates (e.g. regression coefficient) AND variation (e.g. standard deviation) or associated estimates of uncertainty (e.g. confidence intervals)
☐	☑	For null hypothesis testing, the test statistic (e.g. F, t, r) with confidence intervals, effect sizes, degrees of freedom and P value noted<br>*Give P values as exact values whenever suitable.*
☑	☐	For Bayesian analysis, information on the choice of priors and Markov chain Monte Carlo settings
☑	☐	For hierarchical and complex designs, identification of the appropriate level for tests and full reporting of outcomes
☑	☐	Estimates of effect sizes (e.g. Cohen's d, Pearson's r), indicating how they were calculated

Our web collection on statistics for biologists contains articles on many of the points above.

Software and code

Policy information about availability of computer code

Data collection	Data were collected using the electronic Case Report Forms RAVE by Medidata.
Data analysis	Data were analysed using SAS base 9.4 - SAS/STAT 14.3. Only standard methods were used for the analysis as described in the paper.

For manuscripts utilizing custom algorithms or software that are central to the research but not yet described in published literature, software must be made available to editors and reviewers. We strongly encourage code deposition in a community repository (e.g. GitHub). See the Nature Portfolio guidelines for submitting code & software for further information.

Data

Policy information about availability of data

All manuscripts must include a data availability statement. This statement should provide the following information, where applicable:

- Accession codes, unique identifiers, or web links for publicly available datasets
- A description of any restrictions on data availability
- For clinical datasets or third party data, please ensure that the statement adheres to our policy

Availability of the data underlying this publication will be determined according to Bayer's commitment to the EFPIA/PhRMA "Principles for responsible clinical trial data sharing". This pertains to the scope, timepoint and process of data access. As such, Bayer commits to sharing clinical trial data upon request from qualified research personnel at the patient and study level, as well as protocols from clinical trials for medicines and indications approved in the United States (US) and European Union (EU) necessary for conducting legitimate research. This applies to data on new medicines and indications that have been approved by the EU and US regulatory agencies on or after January 01, 2014.

Interested researchers can use www.vivli.org to request access to anonymized patient-level data and supporting documents from clinical studies to conduct further research that can help advance medical science or improve patient care. Information on the Bayer criteria for listing studies and other relevant information is provided in the member section of the portal.

Data access will be granted to anonymized patient-level data, protocols and clinical study reports after approval by an independent scientific review panel. Data will be made available within 6 months after signing the Data Use Agreement to researchers who provide a methodologically sound proposal. Bayer is not involved in the decisions made by the independent review panel. Bayer will take all necessary measures to ensure that patient privacy is safeguarded.

Research involving human participants, their data, or biological material

Policy information about studies with human participants or human data. See also policy information about sex, gender (identity/presentation), and sexual orientation and race, ethnicity and racism.

Reporting on sex and gender	Data from females and males were aggregated and not reported separately
Reporting on race, ethnicity, or other socially relevant groupings	Baseline demographic data present ethnicity (% white) and geographical region.
Population characteristics	Lower-dose osocimab: n = 232; median (range) age, 61.0 (28–91) years; sex (male, %), 61.6<br>Higher-dose osocimab: n = 224; median (range) age, 61.0 (25–90) years; sex (male, %), 63.8<br>Placebo: n = 230; median (range) age, 60.0 years; sex (male, %), 65.2
Recruitment	Participants were identified and recruited according to standard industry guidelines and regulations. Research staff at study sites reviewed their rosters of patients with kidney disease undergoing hemodialysis and approached those who met the inclusion criteria to determine their interest in participation in the trial. Interested patients without exclusion criteria provided signed informed consent prior to performing any study specific testing or procedures. After screening, patients were either randomized or identified as screen failures. Recruitment materials for the study included pocket reference cards and posters for study site personnel and a patient welcome booklet. Recruited patients reflected the age, sex, and racial makeup of the rosters of patients at the various dialysis centers, which may have limited the diversity of those included.
Ethics oversight	An Institutional Review Board at each participating center approved the protocol and all participants provided informed consent. This study was conducted in accordance with the consensus ethical principles derived from international guidelines including the Declaration of Helsinki and Council for International Organizations of Medical Sciences (CIOMS) International Ethical Guidelines, applicable ICH Good Clinical Practice Guidelines and all applicable laws and regulations. A list of all IEC and IRBs is provided in supplementary material.

Note that full information on the approv

Field-specific reporting

Please select the one below that is the best fit for your research. If you are not sure, read the appropriate sections before making your selection.

☑ Life sciences ☐ Behavioural & social sciences ☐ Ecological, evolutionary & environmental sciences

For a reference copy of the document with all sections, see nature.com/documents/nr-reporting-summary-flat.pdf

Life sciences study design

All studies must disclose on these points even when the disclosure is negative.

Sample size	A total of 704 participants from 147 sites in 19 countries were randomized.
Data exclusions	18 participants who did not receive study drug were excluded.
Replication	All pharmacokinetic and pharmacodynamic assays were performed in duplicate at multiple time points.
Randomization	Participants were centrally assigned in a 1:1:1 ratio to lower- or higher-dose osocimab, or to placebo using an interactive web-response system and covariate-adaptive randomization.
Blinding	This was a double-blind trial. The Steering Committee was blinded to treatment assignment as were the members of a Central Independent Adjudication Committee, who adjudicated all deaths, suspected bleeding, and cardiovascular or thromboembolic events.

Reporting for specific materials, systems and methods

We require information from authors about some types of materials, experimental systems and methods used in many studies. Here, indicate whether each material, system or method listed is relevant to your study. If you are not sure if a list item applies to your research, read the appropriate section before selecting a response.

Materials & experimental systems

n/a	Involved in the study	
☑	☐	Antibodies
☑	☐	Eukaryotic cell lines
☑	☐	Palaeontology and archaeology
☑	☐	Animals and other organisms
☐	☑	Clinical dataDual use research of concern
☑	☐	Plants
☑	☐	

Methods

n/a	Involved in the study	
☑	☐	ChIP-seq
☑	☐	Flow cytometry
☑	☐	MRI-based neuroimaging

Ethics oversight	An Institutional Review Board at each participating center approved the protocol and all participants provided informed consent.

Note that full information on the approval of the study protocol must also be provided in the manuscript.

Clinical data

Policy information about clinical studies

All manuscripts should comply with the ICMJE guidelines for publication of clinical research and a completed CONSORT checklist must be included with all submissions.

Clinical trial registration	NCT04523220
Study protocol	Study protocol and SAP provided
Data collection	The sponsor was responsible for data collection, maintenance, and analysis. Data were collected with electronic Case Report Forms (RAVE by Medidata).
Outcomes	The primary outcomes were (1) clinically relevant bleeding, namely the composite of major and clinically relevant nonmajor bleeding, and (2) the composite of moderate or severe adverse events and serious adverse events.

Bleeding was classified as major if it was overt and associated with a decrease in hemoglobin of 2 g/dL or more; necessitated transfusion of two or more units of blood; occurred in a critical area or organ; or contributed to death. Overt bleeding not meeting these criteria, but that necessitated medical examination or intervention, or had clinical consequences, was classified as clinically relevant nonmajor bleeding. If neither set of criteria was met, bleeding was classified as minor.

Prespecified Ssecondary outcomes were assessments of the change from baseline in osocimab concentration and key pharmacodynamic parameters, namely activated partial thromboplastin time and inhibition of factor XIa. Osocimab concentrations were measured by immunoassay, activated partial thromboplastin times were measured using C.K. Prest®, a kaolin activator (Diagnostica Stago, France), and factor XIa inhibition was quantified using a proprietary fluorogenic assay. Assays were conducted after in vitro neutralization of heparin to eliminate the potential effects of heparin in these assays.

Prespecified Eexploratory outcomes included the incidence of major adverse vascular events, the composite of vascular death due to myocardial infarction, stroke, or pulmonary or systemic embolism; nonfatal myocardial infarction or stroke; major amputation of vascular etiology; acute limb ischemia, and symptomatic venous thromboembolism.

Additionally, the incidence of arteriovenous fistula or graft thrombosis, and clotting of the dialysis circuit was assessed at every study visit as a prespecified exploratory outcome. Dialysis circuit clotting in the filter and air trap was assessed at the end of the hemodialysis procedure by study personnel blinded to treatment allocation. S. Clotting scores were assigned using a visual scoring system (0, no clot; 1, trace of clot; 2, intermediate between 1 and 3; and 3, fully clotted system necessitating interruption of hemodialysis session).

This checklist template is licensed under a Creative Commons Attribution 4.0 International License, which permits use, sharing, adaptation, distribution and reproduction in any medium or format, as long as you give appropriate credit to the original author(s) and the source, provide a link to the Creative Commons license, and indicate if changes were made. The images or other third party material in this article are included in the article's Creative Commons license, unless indicated otherwise in a credit line to the material. If material is not included in the article's Creative Commons license and your intended use is not permitted by statutory regulation or exceeds the permitted use, you will need to obtain permission directly from the copyright holder. To view a copy of this license, visit http://creativecommons.org/licenses/by/4.0/
